# Supplementary material for: Sleep disturbance after acute coronary syndrome: A longitudinal study over 12 months
Source: PLoS One. 2022 Jun 3;17(6):e0269545. doi: 10.1371/journal.pone.0269545 (PMC9165780; doi:10.1371/journal.pone.0269545)
Supplement: S1 Dataset — (DOCX) [file pone.0269545.s001.docx]

| Participant number | Time 1=Baseline, 2=3 months 3=12 months | JSS-4 total score | Age | Sex  0=male  1=female | Living status  0=with someone  1=alone | Highest level of education  1=low  2=medium  3=high | Working status  1=full time  2= part time  3=no employment/ retired | MI type  0=non-STEMI  1=STEMI |
| --- | --- | --- | --- | --- | --- | --- | --- | --- |
| 2 | 1 | .00 | 37 | 0 | 2 | 2 | 1 | 0 |
| 2 | 2 | .00 | 37 | 0 | 2 | 2 | 1 | 0 |
| 2 | 3 | 1.00 | 37 | 0 | 2 | 2 | 1 | 0 |
| 3 | 1 | 11.00 | 56 | 1 | 2 | 2 | 2 | 0 |
| 3 | 2 | 14.00 | 56 | 1 | 2 | 2 | 2 | 0 |
| 3 | 3 | 20.00 | 56 | 1 | 2 | 2 | 2 | 0 |
| 4 | 1 | 8.00 | 47 | 0 | 1 | 2 | 1 | 0 |
| 4 | 2 | 5.00 | 47 | 0 | 1 | 2 | 1 | 0 |
| 4 | 3 |  | 47 | 0 | 1 | 2 | 1 | 0 |
| 5 | 1 | .00 | 54 | 0 | 2 | 2 | 1 | 1 |
| 5 | 2 | 3.00 | 54 | 0 | 2 | 2 | 1 | 1 |
| 5 | 3 | 2.00 | 54 | 0 | 2 | 2 | 1 | 1 |
| 6 | 1 | 6.00 | 60 | 1 | 1 | 1 | 3 | 0 |
| 6 | 2 | 10.00 | 60 | 1 | 1 | 1 | 3 | 0 |
| 6 | 3 | 5.00 | 60 | 1 | 1 | 1 | 3 | 0 |
| 7 | 1 | 3.00 | 54 | 0 | 2 | 3 | 1 | 1 |
| 7 | 2 | 1.00 | 54 | 0 | 2 | 3 | 1 | 1 |
| 7 | 3 | .00 | 54 | 0 | 2 | 3 | 1 | 1 |
| 8 | 1 | 7.00 | 50 | 0 | 2 | 2 | 1 | 1 |
| 8 | 2 | 4.00 | 50 | 0 | 2 | 2 | 1 | 1 |
| 8 | 3 | 1.00 | 50 | 0 | 2 | 2 | 1 | 1 |
| 9 | 1 | 6.00 | 65 | 0 | 2 | 2 | 3 | 1 |
| 9 | 2 | 6.00 | 65 | 0 | 2 | 2 | 3 | 1 |
| 9 | 3 | .00 | 65 | 0 | 2 | 2 | 3 | 1 |
| 10 | 1 | 5.00 | 65 | 0 | 2 | 2 | 3 | 0 |
| 10 | 2 | .00 | 65 | 0 | 2 | 2 | 3 | 0 |
| 10 | 3 | 3.00 | 65 | 0 | 2 | 2 | 3 | 0 |
| 11 | 1 | 8.00 | 60 | 0 | 2 | 2 | 3 | 0 |
| 11 | 2 | 11.00 | 60 | 0 | 2 | 2 | 3 | 0 |
| 11 | 3 | .00 | 60 | 0 | 2 | 2 | 3 | 0 |
| 12 | 1 | 20.00 | 49 | 0 | 2 | 3 | 1 | 1 |
| 12 | 2 |  | 49 | 0 | 2 | 3 | 1 | 1 |
| 12 | 3 |  | 49 | 0 | 2 | 3 | 1 | 1 |
| 13 | 1 | 7.00 | 65 | 0 | 2 | 1 | 3 | 1 |
| 13 | 2 |  | 65 | 0 | 2 | 1 | 3 | 1 |
| 13 | 3 |  | 65 | 0 | 2 | 1 | 3 | 1 |
| 14 | 1 | 10.00 | 62 | 0 | 2 | 1 | 3 | 0 |
| 14 | 2 | .00 | 62 | 0 | 2 | 1 | 3 | 0 |
| 14 | 3 | 3.00 | 62 | 0 | 2 | 1 | 3 | 0 |
| 16 | 1 | 2.00 | 62 | 1 | 2 | 1 | 3 | 0 |
| 16 | 2 |  | 62 | 1 | 2 | 1 | 3 | 0 |
| 16 | 3 |  | 62 | 1 | 2 | 1 | 3 | 0 |
| 17 | 1 | 7.00 | 63 | 0 | 2 | 2 | 1 | 1 |
| 17 | 2 | 1.00 | 63 | 0 | 2 | 2 | 1 | 1 |
| 17 | 3 | 4.00 | 63 | 0 | 2 | 2 | 1 | 1 |
| 18 | 1 | .00 | 80 | 0 | 2 | 2 | 3 | 1 |
| 18 | 2 |  | 80 | 0 | 2 | 2 | 3 | 1 |
| 18 | 3 |  | 80 | 0 | 2 | 2 | 3 | 1 |
| 19 | 1 | 1.00 | 63 | 0 | 2 | 2 | 1 | 1 |
| 19 | 2 | .00 | 63 | 0 | 2 | 2 | 1 | 1 |
| 19 | 3 | .00 | 63 | 0 | 2 | 2 | 1 | 1 |
| 21 | 1 | 20.00 | 62 | 0 | 1 | 1 | 3 | 0 |
| 21 | 2 | 20.00 | 62 | 0 | 1 | 1 | 3 | 0 |
| 21 | 3 | 5.00 | 62 | 0 | 1 | 1 | 3 | 0 |
| 22 | 1 | 4.00 | 55 | 0 | 2 | 2 | 2 | 0 |
| 22 | 2 | 1.00 | 55 | 0 | 2 | 2 | 2 | 0 |
| 22 | 3 | 3.00 | 55 | 0 | 2 | 2 | 2 | 0 |
| 24 | 1 | .00 | 61 | 1 | 1 | 3 | 2 | 1 |
| 24 | 2 | .00 | 61 | 1 | 1 | 3 | 2 | 1 |
| 24 | 3 | .00 | 61 | 1 | 1 | 3 | 2 | 1 |
| 25 | 1 | 6.00 | 64 | 1 | 2 | 3 | 3 | 0 |
| 25 | 2 | 2.00 | 64 | 1 | 2 | 3 | 3 | 0 |
| 25 | 3 | 6.00 | 64 | 1 | 2 | 3 | 3 | 0 |
| 26 | 1 | .00 | 75 | 0 | 1 | 2 | 3 | 1 |
| 26 | 2 | .00 | 75 | 0 | 1 | 2 | 3 | 1 |
| 26 | 3 | .00 | 75 | 0 | 1 | 2 | 3 | 1 |
| 27 | 1 | 5.00 | 56 | 0 | 2 | 1 | 2 | 0 |
| 27 | 2 |  | 56 | 0 | 2 | 1 | 2 | 0 |
| 27 | 3 |  | 56 | 0 | 2 | 1 | 2 | 0 |
| 28 | 1 | 11.00 | 63 | 0 | 1 | 3 | 2 | 0 |
| 28 | 2 | 4.00 | 63 | 0 | 1 | 3 | 2 | 0 |
| 28 | 3 | 2.00 | 63 | 0 | 1 | 3 | 2 | 0 |
| 29 | 1 | .00 | 45 | 0 | 2 | 2 | 1 | 0 |
| 29 | 2 | 5.00 | 45 | 0 | 2 | 2 | 1 | 0 |
| 29 | 3 | 5.00 | 45 | 0 | 2 | 2 | 1 | 0 |
| 30 | 1 | .00 | 66 | 0 | 2 | 2 | 2 | 1 |
| 30 | 2 | 2.00 | 66 | 0 | 2 | 2 | 2 | 1 |
| 30 | 3 | 2.00 | 66 | 0 | 2 | 2 | 2 | 1 |
| 31 | 1 | 6.00 | 49 | 0 | 2 | 2 | 1 | 0 |
| 31 | 2 | .00 | 49 | 0 | 2 | 2 | 1 | 0 |
| 31 | 3 | .00 | 49 | 0 | 2 | 2 | 1 | 0 |
| 32 | 1 | 4.00 | 53 | 0 | 2 | 2 | 2 | 0 |
| 32 | 2 | 3.00 | 53 | 0 | 2 | 2 | 2 | 0 |
| 32 | 3 | 5.00 | 53 | 0 | 2 | 2 | 2 | 0 |
| 33 | 1 | 4.00 | 78 | 0 | 1 | 3 | 3 | 0 |
| 33 | 2 |  | 78 | 0 | 1 | 3 | 3 | 0 |
| 33 | 3 |  | 78 | 0 | 1 | 3 | 3 | 0 |
| 34 | 1 | 15.00 | 57 | 1 | 1 | 2 | 3 | 0 |
| 34 | 2 | 12.00 | 57 | 1 | 1 | 2 | 3 | 0 |
| 34 | 3 | 10.00 | 57 | 1 | 1 | 2 | 3 | 0 |
| 35 | 1 | 1.00 | 44 | 0 | 1 | 2 | 1 | 0 |
| 35 | 2 | 1.00 | 44 | 0 | 1 | 2 | 1 | 0 |
| 35 | 3 |  | 44 | 0 | 1 | 2 | 1 | 0 |
| 36 | 1 | 13.00 | 57 | 0 | 2 | 3 | 3 | 1 |
| 36 | 2 |  | 57 | 0 | 2 | 3 | 3 | 1 |
| 36 | 3 |  | 57 | 0 | 2 | 3 | 3 | 1 |
| 37 | 1 | 15.00 | 78 | 1 | 1 | 2 | 3 | 1 |
| 37 | 2 |  | 78 | 1 | 1 | 2 | 3 | 1 |
| 37 | 3 |  | 78 | 1 | 1 | 2 | 3 | 1 |
| 38 | 1 | 8.00 | 47 | 0 | 2 | 2 | 1 | 0 |
| 38 | 2 | 16.00 | 47 | 0 | 2 | 2 | 1 | 0 |
| 38 | 3 | 10.00 | 47 | 0 | 2 | 2 | 1 | 0 |
| 39 | 1 | 4.00 | 63 | 1 | 2 | 2 | 2 | 0 |
| 39 | 2 | 15.00 | 63 | 1 | 2 | 2 | 2 | 0 |
| 39 | 3 | 2.00 | 63 | 1 | 2 | 2 | 2 | 0 |
| 40 | 1 | 5.00 | 70 | 0 | 2 | 1 | 3 | 0 |
| 40 | 2 | 3.00 | 70 | 0 | 2 | 1 | 3 | 0 |
| 40 | 3 | 5.00 | 70 | 0 | 2 | 1 | 3 | 0 |
| 41 | 1 | 6.00 | 51 | 0 | 2 | 1 | 1 | 1 |
| 41 | 2 | .00 | 51 | 0 | 2 | 1 | 1 | 1 |
| 41 | 3 | 5.00 | 51 | 0 | 2 | 1 | 1 | 1 |
| 42 | 1 | 8.00 | 60 | 1 | 1 | 2 | 2 | 0 |
| 42 | 2 | 11.00 | 60 | 1 | 1 | 2 | 2 | 0 |
| 42 | 3 | 8.00 | 60 | 1 | 1 | 2 | 2 | 0 |
| 43 | 1 | 5.00 | 68 | 0 | 2 | 2 | 3 | 0 |
| 43 | 2 | 8.00 | 68 | 0 | 2 | 2 | 3 | 0 |
| 43 | 3 | 5.00 | 68 | 0 | 2 | 2 | 3 | 0 |
| 44 | 1 | 13.00 | 64 | 0 | 2 | 2 | 3 | 1 |
| 44 | 2 | 9.00 | 64 | 0 | 2 | 2 | 3 | 1 |
| 44 | 3 | 2.00 | 64 | 0 | 2 | 2 | 3 | 1 |
| 45 | 1 | 4.00 | 64 | 0 | 2 | 2 | 3 | 0 |
| 45 | 2 | .00 | 64 | 0 | 2 | 2 | 3 | 0 |
| 45 | 3 | 2.00 | 64 | 0 | 2 | 2 | 3 | 0 |
| 46 | 1 | 6.00 | 73 | 0 | 2 | 2 | 3 | 1 |
| 46 | 2 | 2.00 | 73 | 0 | 2 | 2 | 3 | 1 |
| 46 | 3 | 6.00 | 73 | 0 | 2 | 2 | 3 | 1 |
| 47 | 1 | 9.00 | 51 | 1 | 1 | 2 | 2 | 0 |
| 47 | 2 | 9.00 | 51 | 1 | 1 | 2 | 2 | 0 |
| 47 | 3 |  | 51 | 1 | 1 | 2 | 2 | 0 |
| 48 | 1 | 1.00 | 44 | 0 | 2 | 2 | 1 | 1 |
| 48 | 2 | .00 | 44 | 0 | 2 | 2 | 1 | 1 |
| 48 | 3 | .00 | 44 | 0 | 2 | 2 | 1 | 1 |
| 49 | 1 | 12.00 | 67 | 0 | 2 | 2 | 3 | 0 |
| 49 | 2 | 7.00 | 67 | 0 | 2 | 2 | 3 | 0 |
| 49 | 3 | 1.00 | 67 | 0 | 2 | 2 | 3 | 0 |
| 50 | 1 | 16.00 | 43 | 0 | 2 | 2 | 1 | 0 |
| 50 | 2 | 16.00 | 43 | 0 | 2 | 2 | 1 | 0 |
| 50 | 3 | 4.00 | 43 | 0 | 2 | 2 | 1 | 0 |
| 51 | 1 | 16.00 | 60 | 0 | 2 | 2 | 1 | 0 |
| 51 | 2 | 13.00 | 60 | 0 | 2 | 2 | 1 | 0 |
| 51 | 3 | 9.00 | 60 | 0 | 2 | 2 | 1 | 0 |
| 52 | 1 | 3.00 | 65 | 0 | 1 | 2 | 3 | 1 |
| 52 | 2 |  | 65 | 0 | 1 | 2 | 3 | 1 |
| 52 | 3 |  | 65 | 0 | 1 | 2 | 3 | 1 |
| 53 | 1 | 8.00 | 69 | 0 | 2 | 3 | 2 | 0 |
| 53 | 2 | 11.00 | 69 | 0 | 2 | 3 | 2 | 0 |
| 53 | 3 | 5.00 | 69 | 0 | 2 | 3 | 2 | 0 |
| 54 | 1 | 3.00 | 48 | 0 | 2 | 2 | 1 | 0 |
| 54 | 2 | 10.00 | 48 | 0 | 2 | 2 | 1 | 0 |
| 54 | 3 | 6.00 | 48 | 0 | 2 | 2 | 1 | 0 |
| 55 | 1 | 12.00 | 56 | 0 | 2 | 3 | 1 | 0 |
| 55 | 2 | 1.00 | 56 | 0 | 2 | 3 | 1 | 0 |
| 55 | 3 | .00 | 56 | 0 | 2 | 3 | 1 | 0 |
| 56 | 1 | 4.00 | 67 | 1 | 1 | 2 | 3 | 0 |
| 56 | 2 | 6.00 | 67 | 1 | 1 | 2 | 3 | 0 |
| 56 | 3 | 6.00 | 67 | 1 | 1 | 2 | 3 | 0 |
| 57 | 1 | 7.00 | 51 | 1 | 2 | 2 | 1 | 0 |
| 57 | 2 | 6.00 | 51 | 1 | 2 | 2 | 1 | 0 |
| 57 | 3 | 6.00 | 51 | 1 | 2 | 2 | 1 | 0 |
| 58 | 1 | 12.00 | 68 | 0 | 1 | 2 | 3 | 0 |
| 58 | 2 | 4.00 | 68 | 0 | 1 | 2 | 3 | 0 |
| 58 | 3 | 9.00 | 68 | 0 | 1 | 2 | 3 | 0 |
| 59 | 1 | 4.00 | 51 | 0 | 2 | 2 | 1 | 1 |
| 59 | 2 | 12.00 | 51 | 0 | 2 | 2 | 1 | 1 |
| 59 | 3 | 11.00 | 51 | 0 | 2 | 2 | 1 | 1 |
| 60 | 1 | .00 | 68 | 0 | 2 | 2 | 3 | 0 |
| 60 | 2 | 5.00 | 68 | 0 | 2 | 2 | 3 | 0 |
| 60 | 3 | 5.00 | 68 | 0 | 2 | 2 | 3 | 0 |
| 61 | 1 | 15.00 | 75 | 0 | 2 | 2 | 3 | 0 |
| 61 | 2 |  | 75 | 0 | 2 | 2 | 3 | 0 |
| 61 | 3 |  | 75 | 0 | 2 | 2 | 3 | 0 |
| 62 | 1 | .00 | 51 | 0 | 1 | 2 | 1 | 1 |
| 62 | 2 |  | 51 | 0 | 1 | 2 | 1 | 1 |
| 62 | 3 |  | 51 | 0 | 1 | 2 | 1 | 1 |
| 63 | 1 | 2.00 | 41 | 0 | 2 | 2 | 1 | 0 |
| 63 | 2 | 5.00 | 41 | 0 | 2 | 2 | 1 | 0 |
| 63 | 3 |  | 41 | 0 | 2 | 2 | 1 | 0 |
| 64 | 1 | 3.00 | 41 | 0 | 2 | 3 | 1 | 1 |
| 64 | 2 | 2.00 | 41 | 0 | 2 | 3 | 1 | 1 |
| 64 | 3 | 1.00 | 41 | 0 | 2 | 3 | 1 | 1 |
| 65 | 1 | 13.00 | 66 | 0 | 2 | 2 | 3 | 0 |
| 65 | 2 | 2.00 | 66 | 0 | 2 | 2 | 3 | 0 |
| 65 | 3 | 7.00 | 66 | 0 | 2 | 2 | 3 | 0 |
| 66 | 1 | 9.00 | 66 | 0 | 1 | 3 | 3 | 0 |
| 66 | 2 | .00 | 66 | 0 | 1 | 3 | 3 | 0 |
| 66 | 3 | 3.00 | 66 | 0 | 1 | 3 | 3 | 0 |
| 67 | 1 | 7.00 | 47 | 0 | 2 | 2 | 1 | 0 |
| 67 | 2 | 8.00 | 47 | 0 | 2 | 2 | 1 | 0 |
| 67 | 3 | 5.00 | 47 | 0 | 2 | 2 | 1 | 0 |
| 68 | 1 | .00 | 62 | 0 | 2 | 2 | 1 | 0 |
| 68 | 2 | 10.00 | 62 | 0 | 2 | 2 | 1 | 0 |
| 68 | 3 | 5.00 | 62 | 0 | 2 | 2 | 1 | 0 |
| 69 | 1 | 11.00 | 70 | 0 | 2 | 2 | 1 | 1 |
| 69 | 2 | 6.00 | 70 | 0 | 2 | 2 | 1 | 1 |
| 69 | 3 |  | 70 | 0 | 2 | 2 | 1 | 1 |
| 72 | 1 | 1.00 | 75 | 0 | 1 | 2 | 3 | 1 |
| 72 | 2 | 1.00 | 75 | 0 | 1 | 2 | 3 | 1 |
| 72 | 3 | 5.00 | 75 | 0 | 1 | 2 | 3 | 1 |
| 73 | 1 | 8.00 | 77 | 0 | 1 | 2 | 3 | 1 |
| 73 | 2 | 9.00 | 77 | 0 | 1 | 2 | 3 | 1 |
| 73 | 3 | .00 | 77 | 0 | 1 | 2 | 3 | 1 |
| 74 | 1 | 4.00 | 74 | 1 | 1 | 1 | 3 | 0 |
| 74 | 2 |  | 74 | 1 | 1 | 1 | 3 | 0 |
| 74 | 3 |  | 74 | 1 | 1 | 1 | 3 | 0 |
| 75 | 1 | 11.00 | 78 | 0 | 2 | 1 | 3 | 1 |
| 75 | 2 |  | 78 | 0 | 2 | 1 | 3 | 1 |
| 75 | 3 |  | 78 | 0 | 2 | 1 | 3 | 1 |
| 76 | 1 | .00 | 88 | 0 | 2 | 2 | 3 | 0 |
| 76 | 2 |  | 88 | 0 | 2 | 2 | 3 | 0 |
| 76 | 3 | 10.00 | 88 | 0 | 2 | 2 | 3 | 0 |
| 77 | 1 | 2.00 | 52 | 0 | 2 | 3 | 1 | 0 |
| 77 | 2 |  | 52 | 0 | 2 | 3 | 1 | 0 |
| 77 | 3 |  | 52 | 0 | 2 | 3 | 1 | 0 |
| 78 | 1 | 10.00 | 59 | 1 | 1 | 2 | 3 | 0 |
| 78 | 2 | 12.00 | 59 | 1 | 1 | 2 | 3 | 0 |
| 78 | 3 | .00 | 59 | 1 | 1 | 2 | 3 | 0 |
| 80 | 1 | 15.00 | 52 | 1 | 1 | 2 | 3 | 0 |
| 80 | 2 |  | 52 | 1 | 1 | 2 | 3 | 0 |
| 80 | 3 |  | 52 | 1 | 1 | 2 | 3 | 0 |
| 81 | 1 | 12.00 | 67 | 1 | 1 | 2 | 3 | 0 |
| 81 | 2 | 7.00 | 67 | 1 | 1 | 2 | 3 | 0 |
| 81 | 3 | 10.00 | 67 | 1 | 1 | 2 | 3 | 0 |
| 82 | 1 | 8.00 | 74 | 0 | 2 | 2 | 3 | 0 |
| 82 | 2 |  | 74 | 0 | 2 | 2 | 3 | 0 |
| 82 | 3 | 6.00 | 74 | 0 | 2 | 2 | 3 | 0 |
| 83 | 1 | 13.00 | 64 | 0 | 2 | 2 | 2 | 1 |
| 83 | 2 | .00 | 64 | 0 | 2 | 2 | 2 | 1 |
| 83 | 3 | 1.00 | 64 | 0 | 2 | 2 | 2 | 1 |
| 84 | 1 | 2.00 | 77 | 1 | 1 | 1 | 3 | 1 |
| 84 | 2 | 6.00 | 77 | 1 | 1 | 1 | 3 | 1 |
| 84 | 3 | 6.00 | 77 | 1 | 1 | 1 | 3 | 1 |
| 85 | 1 | 1.00 | 44 | 0 | 2 | 1 | 3 | 0 |
| 85 | 2 | 1.00 | 44 | 0 | 2 | 1 | 3 | 0 |
| 85 | 3 | 5.00 | 44 | 0 | 2 | 1 | 3 | 0 |
| 86 | 1 | .00 | 49 | 0 | 2 | 1 | 1 | 1 |
| 86 | 2 | 16.00 | 49 | 0 | 2 | 1 | 1 | 1 |
| 86 | 3 | 9.00 | 49 | 0 | 2 | 1 | 1 | 1 |
| 87 | 1 | 2.00 | 64 | 0 | 2 | 2 | 1 | 0 |
| 87 | 2 | 4.00 | 64 | 0 | 2 | 2 | 1 | 0 |
| 87 | 3 | 1.00 | 64 | 0 | 2 | 2 | 1 | 0 |
| 88 | 1 | 8.00 | 60 | 0 | 2 | 3 | 1 | 0 |
| 88 | 2 | 6.00 | 60 | 0 | 2 | 3 | 1 | 0 |
| 88 | 3 | 7.00 | 60 | 0 | 2 | 3 | 1 | 0 |
| 90 | 1 | 14.00 | 47 | 1 | 2 | 2 | 2 | 1 |
| 90 | 2 | 8.00 | 47 | 1 | 2 | 2 | 2 | 1 |
| 90 | 3 | 7.00 | 47 | 1 | 2 | 2 | 2 | 1 |
| 91 | 1 | 7.00 | 73 | 0 | 2 | 3 | 3 | 0 |
| 91 | 2 | 1.00 | 73 | 0 | 2 | 3 | 3 | 0 |
| 91 | 3 | .00 | 73 | 0 | 2 | 3 | 3 | 0 |
| 92 | 1 | 8.00 | 50 | 1 | 2 | 1 | 1 | 0 |
| 92 | 2 | 8.00 | 50 | 1 | 2 | 1 | 1 | 0 |
| 92 | 3 | 14.00 | 50 | 1 | 2 | 1 | 1 | 0 |
| 93 | 1 | .00 | 46 | 0 | 2 | 2 | 1 | 0 |
| 93 | 2 | 1.00 | 46 | 0 | 2 | 2 | 1 | 0 |
| 93 | 3 | .00 | 46 | 0 | 2 | 2 | 1 | 0 |
| 94 | 1 | .00 | 52 | 0 | 2 | 2 | 1 | 0 |
| 94 | 2 | .00 | 52 | 0 | 2 | 2 | 1 | 0 |
| 94 | 3 | .00 | 52 | 0 | 2 | 2 | 1 | 0 |
| 95 | 1 | 2.00 | 55 | 0 | 2 | 2 | 1 | 0 |
| 95 | 2 | 1.00 | 55 | 0 | 2 | 2 | 1 | 0 |
| 95 | 3 |  | 55 | 0 | 2 | 2 | 1 | 0 |
| 96 | 1 | 20.00 | 57 | 1 | 1 | 2 | 3 | 0 |
| 96 | 2 |  | 57 | 1 | 1 | 2 | 3 | 0 |
| 96 | 3 |  | 57 | 1 | 1 | 2 | 3 | 0 |
| 97 | 1 | 3.00 | 59 | 0 | 2 | 2 | 1 | 0 |
| 97 | 2 | 6.00 | 59 | 0 | 2 | 2 | 1 | 0 |
| 97 | 3 | 5.00 | 59 | 0 | 2 | 2 | 1 | 0 |
| 98 | 1 | 5.00 | 59 | 0 | 2 | 3 | 1 | 0 |
| 98 | 2 | .00 | 59 | 0 | 2 | 3 | 1 | 0 |
| 98 | 3 | 4.00 | 59 | 0 | 2 | 3 | 1 | 0 |
| 99 | 1 | 2.00 | 60 | 0 | 2 | 2 | 1 | 0 |
| 99 | 2 | 1.00 | 60 | 0 | 2 | 2 | 1 | 0 |
| 99 | 3 | 2.00 | 60 | 0 | 2 | 2 | 1 | 0 |
| 100 | 1 | .00 | 57 | 0 | 2 | 2 | 1 | 0 |
| 100 | 2 | 1.00 | 57 | 0 | 2 | 2 | 1 | 0 |
| 100 | 3 | 2.00 | 57 | 0 | 2 | 2 | 1 | 0 |
| 101 | 1 | 5.00 | 57 | 0 | 2 | 3 | 1 | 0 |
| 101 | 2 | 6.00 | 57 | 0 | 2 | 3 | 1 | 0 |
| 101 | 3 | 7.00 | 57 | 0 | 2 | 3 | 1 | 0 |
| 102 | 1 | 1.00 | 60 | 0 | 2 | 2 | 3 | 0 |
| 102 | 2 | 6.00 | 60 | 0 | 2 | 2 | 3 | 0 |
| 102 | 3 | 5.00 | 60 | 0 | 2 | 2 | 3 | 0 |
| 103 | 1 | 1.00 | 79 | 1 | 1 | 3 | 3 | 1 |
| 103 | 2 |  | 79 | 1 | 1 | 3 | 3 | 1 |
| 103 | 3 |  | 79 | 1 | 1 | 3 | 3 | 1 |
| 104 | 1 | 13.00 | 75 | 1 | 1 | 2 | 3 | 1 |
| 104 | 2 | 13.00 | 75 | 1 | 1 | 2 | 3 | 1 |
| 104 | 3 | 10.00 | 75 | 1 | 1 | 2 | 3 | 1 |
| 105 | 1 | 14.00 | 53 | 0 | 2 | 2 | 1 | 0 |
| 105 | 2 |  | 53 | 0 | 2 | 2 | 1 | 0 |
| 105 | 3 |  | 53 | 0 | 2 | 2 | 1 | 0 |
| 106 | 1 | 6.00 | 59 | 0 | 2 | 2 | 1 | 0 |
| 106 | 2 | 6.00 | 59 | 0 | 2 | 2 | 1 | 0 |
| 106 | 3 | 2.00 | 59 | 0 | 2 | 2 | 1 | 0 |
| 107 | 1 | 5.00 | 68 | 1 | 2 | 3 | 3 | 0 |
| 107 | 2 | 12.00 | 68 | 1 | 2 | 3 | 3 | 0 |
| 107 | 3 | 4.00 | 68 | 1 | 2 | 3 | 3 | 0 |
| 108 | 1 | 12.00 | 38 | 0 | 1 | 2 | 1 | 0 |
| 108 | 2 | 13.00 | 38 | 0 | 1 | 2 | 1 | 0 |
| 108 | 3 | 3.00 | 38 | 0 | 1 | 2 | 1 | 0 |
| 109 | 1 | 7.00 | 47 | 0 | 1 | 2 | 1 | 0 |
| 109 | 2 | 9.00 | 47 | 0 | 1 | 2 | 1 | 0 |
| 109 | 3 | 2.00 | 47 | 0 | 1 | 2 | 1 | 0 |
| 110 | 1 | .00 | 70 | 0 | 2 | 2 | 3 | 0 |
| 110 | 2 |  | 70 | 0 | 2 | 2 | 3 | 0 |
| 110 | 3 |  | 70 | 0 | 2 | 2 | 3 | 0 |
| 111 | 1 | .00 | 45 | 0 | 2 | 3 | 1 | 0 |
| 111 | 2 | 1.00 | 45 | 0 | 2 | 3 | 1 | 0 |
| 111 | 3 | .00 | 45 | 0 | 2 | 3 | 1 | 0 |
| 112 | 1 | 7.00 | 54 | 0 | 2 | 2 | 1 | 0 |
| 112 | 2 | 14.00 | 54 | 0 | 2 | 2 | 1 | 0 |
| 112 | 3 |  | 54 | 0 | 2 | 2 | 1 | 0 |
| 113 | 1 | 4.00 | 64 | 0 | 1 | 2 | 1 | 0 |
| 113 | 2 | .00 | 64 | 0 | 1 | 2 | 1 | 0 |
| 113 | 3 | .00 | 64 | 0 | 1 | 2 | 1 | 0 |
| 114 | 1 | 5.00 | 57 | 0 | 2 | 1 | 1 | 1 |
| 114 | 2 | 4.00 | 57 | 0 | 2 | 1 | 1 | 1 |
| 114 | 3 | 20.00 | 57 | 0 | 2 | 1 | 1 | 1 |
| 115 | 1 | 2.00 | 51 | 0 | 2 | 2 | 1 | 1 |
| 115 | 2 | 9.00 | 51 | 0 | 2 | 2 | 1 | 1 |
| 115 | 3 | 6.00 | 51 | 0 | 2 | 2 | 1 | 1 |
| 116 | 1 | 18.00 | 54 | 0 | 2 | 2 | 1 | 0 |
| 116 | 2 | 15.00 | 54 | 0 | 2 | 2 | 1 | 0 |
| 116 | 3 | 15.00 | 54 | 0 | 2 | 2 | 1 | 0 |
| 117 | 1 | 1.00 | 62 | 0 | 1 | 2 | 1 | 1 |
| 117 | 2 | 10.00 | 62 | 0 | 1 | 2 | 1 | 1 |
| 117 | 3 | 15.00 | 62 | 0 | 1 | 2 | 1 | 1 |
| 118 | 1 | .00 | 81 | 1 | 1 | 2 | 3 | 1 |
| 118 | 2 |  | 81 | 1 | 1 | 2 | 3 | 1 |
| 118 | 3 |  | 81 | 1 | 1 | 2 | 3 | 1 |
| 119 | 1 | 20.00 | 64 | 0 | 2 | 2 | 3 | 0 |
| 119 | 2 | 1.00 | 64 | 0 | 2 | 2 | 3 | 0 |
| 119 | 3 | 6.00 | 64 | 0 | 2 | 2 | 3 | 0 |
| 120 | 1 | 10.00 | 59 | 0 | 2 | 2 | 1 | 1 |
| 120 | 2 | 12.00 | 59 | 0 | 2 | 2 | 1 | 1 |
| 120 | 3 | 7.00 | 59 | 0 | 2 | 2 | 1 | 1 |
| 121 | 1 | 7.00 | 52 | 0 | 2 | 2 | 1 | 1 |
| 121 | 2 | 1.00 | 52 | 0 | 2 | 2 | 1 | 1 |
| 121 | 3 | .00 | 52 | 0 | 2 | 2 | 1 | 1 |
| 122 | 1 | 2.00 | 62 | 0 | 2 | 2 | 1 | 0 |
| 122 | 2 |  | 62 | 0 | 2 | 2 | 1 | 0 |
| 122 | 3 |  | 62 | 0 | 2 | 2 | 1 | 0 |
| 123 | 1 | .00 | 58 | 0 | 2 | 3 | 1 | 0 |
| 123 | 2 | 2.00 | 58 | 0 | 2 | 3 | 1 | 0 |
| 123 | 3 | 4.00 | 58 | 0 | 2 | 3 | 1 | 0 |
| 124 | 1 | .00 | 54 | 0 | 2 | 2 | 3 | 0 |
| 124 | 2 |  | 54 | 0 | 2 | 2 | 3 | 0 |
| 124 | 3 |  | 54 | 0 | 2 | 2 | 3 | 0 |
| 125 | 1 | .00 | 57 | 0 | 2 | 2 | 1 | 0 |
| 125 | 2 | 7.00 | 57 | 0 | 2 | 2 | 1 | 0 |
| 125 | 3 | 7.00 | 57 | 0 | 2 | 2 | 1 | 0 |
| 126 | 1 | 5.00 | 77 | 0 | 2 | 2 | 3 | 0 |
| 126 | 2 | 10.00 | 77 | 0 | 2 | 2 | 3 | 0 |
| 126 | 3 | 10.00 | 77 | 0 | 2 | 2 | 3 | 0 |
| 127 | 1 | 8.00 | 73 | 0 | 2 | 2 | 3 | 0 |
| 127 | 2 | .00 | 73 | 0 | 2 | 2 | 3 | 0 |
| 127 | 3 | .00 | 73 | 0 | 2 | 2 | 3 | 0 |
| 128 | 1 | .00 | 65 | 0 | 2 | 3 | 3 | 0 |
| 128 | 2 | 7.00 | 65 | 0 | 2 | 3 | 3 | 0 |
| 128 | 3 | 6.00 | 65 | 0 | 2 | 3 | 3 | 0 |
| 129 | 1 | 12.00 | 58 | 0 | 2 | 2 | 1 | 0 |
| 129 | 2 | 1.00 | 58 | 0 | 2 | 2 | 1 | 0 |
| 129 | 3 | 4.00 | 58 | 0 | 2 | 2 | 1 | 0 |
| 130 | 1 | 2.00 | 55 | 0 | 2 | 3 | 2 | 0 |
| 130 | 2 | 4.00 | 55 | 0 | 2 | 3 | 2 | 0 |
| 130 | 3 | 8.00 | 55 | 0 | 2 | 3 | 2 | 0 |
| 131 | 1 | 5.00 | 59 | 0 | 2 | 2 | 1 | 0 |
| 131 | 2 | .00 | 59 | 0 | 2 | 2 | 1 | 0 |
| 131 | 3 | .00 | 59 | 0 | 2 | 2 | 1 | 0 |
| 132 | 1 | 3.00 | 62 | 0 | 2 | 2 | 3 | 0 |
| 132 | 2 | .00 | 62 | 0 | 2 | 2 | 3 | 0 |
| 132 | 3 | .00 | 62 | 0 | 2 | 2 | 3 | 0 |
| 133 | 1 | 2.00 | 47 | 1 | 2 | 2 | 2 | 1 |
| 133 | 2 | 4.00 | 47 | 1 | 2 | 2 | 2 | 1 |
| 133 | 3 | .00 | 47 | 1 | 2 | 2 | 2 | 1 |
| 134 | 1 | 12.00 | 50 | 1 | 2 | 3 | 1 | 1 |
| 134 | 2 | 5.00 | 50 | 1 | 2 | 3 | 1 | 1 |
| 134 | 3 | 13.00 | 50 | 1 | 2 | 3 | 1 | 1 |
| 135 | 1 | .00 | 61 | 0 | 1 | 2 | 2 | 1 |
| 135 | 2 | 5.00 | 61 | 0 | 1 | 2 | 2 | 1 |
| 135 | 3 | 5.00 | 61 | 0 | 1 | 2 | 2 | 1 |
| 136 | 1 | 4.00 | 55 | 0 | 2 | 3 | 1 | 0 |
| 136 | 2 | .00 | 55 | 0 | 2 | 3 | 1 | 0 |
| 136 | 3 | .00 | 55 | 0 | 2 | 3 | 1 | 0 |
| 137 | 1 | .00 | 49 | 0 | 2 | 2 | 1 | 0 |
| 137 | 2 | .00 | 49 | 0 | 2 | 2 | 1 | 0 |
| 137 | 3 |  | 49 | 0 | 2 | 2 | 1 | 0 |
| 138 | 1 | 6.00 | 51 | 0 | 1 | 2 | 1 | 0 |
| 138 | 2 | 1.00 | 51 | 0 | 1 | 2 | 1 | 0 |
| 138 | 3 | 2.00 | 51 | 0 | 1 | 2 | 1 | 0 |
| 139 | 1 | 10.00 | 57 | 0 | 2 | 2 | 2 | 0 |
| 139 | 2 |  | 57 | 0 | 2 | 2 | 2 | 0 |
| 139 | 3 |  | 57 | 0 | 2 | 2 | 2 | 0 |
| 140 | 1 | 8.00 | 78 | 0 | 2 | 2 | 3 | 0 |
| 140 | 2 | 3.00 | 78 | 0 | 2 | 2 | 3 | 0 |
| 140 | 3 | 4.00 | 78 | 0 | 2 | 2 | 3 | 0 |
| 141 | 1 | 9.00 | 84 | 0 | 2 | 2 | 3 | 1 |
| 141 | 2 | .00 | 84 | 0 | 2 | 2 | 3 | 1 |
| 141 | 3 |  | 84 | 0 | 2 | 2 | 3 | 1 |
| 142 | 1 | 7.00 | 59 | 0 | 2 | 2 | 1 | 0 |
| 142 | 2 | 12.00 | 59 | 0 | 2 | 2 | 1 | 0 |
| 142 | 3 |  | 59 | 0 | 2 | 2 | 1 | 0 |
| 143 | 1 | 16.00 | 39 | 0 | 2 | 2 | 2 | 0 |
| 143 | 2 | 6.00 | 39 | 0 | 2 | 2 | 2 | 0 |
| 143 | 3 |  | 39 | 0 | 2 | 2 | 2 | 0 |
| 144 | 1 | 2.00 | 45 | 0 | 2 | 2 | 1 | 0 |
| 144 | 2 | .00 | 45 | 0 | 2 | 2 | 1 | 0 |
| 144 | 3 |  | 45 | 0 | 2 | 2 | 1 | 0 |
| 145 | 1 | 1.00 | 54 | 0 | 2 | 2 | 1 | 0 |
| 145 | 2 | 1.00 | 54 | 0 | 2 | 2 | 1 | 0 |
| 145 | 3 |  | 54 | 0 | 2 | 2 | 1 | 0 |
| 146 | 1 | 2.00 | 58 | 0 | 2 | 2 | 1 | 1 |
| 146 | 2 | .00 | 58 | 0 | 2 | 2 | 1 | 1 |
| 146 | 3 |  | 58 | 0 | 2 | 2 | 1 | 1 |
| 147 | 1 | 1.00 | 84 | 0 | 2 | 2 | 3 | 0 |
| 147 | 2 | .00 | 84 | 0 | 2 | 2 | 3 | 0 |
| 147 | 3 |  | 84 | 0 | 2 | 2 | 3 | 0 |
| 148 | 1 | 18.00 | 59 | 0 | 1 | 1 | 3 | 0 |
| 148 | 2 |  | 59 | 0 | 1 | 1 | 3 | 0 |
| 148 | 3 |  | 59 | 0 | 1 | 1 | 3 | 0 |
| 149 | 1 | 10.00 | 68 | 0 | 1 | 2 | 3 | 0 |
| 149 | 2 | 2.00 | 68 | 0 | 1 | 2 | 3 | 0 |
| 149 | 3 |  | 68 | 0 | 1 | 2 | 3 | 0 |
| 150 | 1 | 5.00 | 51 | 0 | 1 | 2 | 1 | 0 |
| 150 | 2 | 3.00 | 51 | 0 | 1 | 2 | 1 | 0 |
| 150 | 3 |  | 51 | 0 | 1 | 2 | 1 | 0 |
| 151 | 1 | 7.00 | 76 | 0 | 2 | 3 | 3 | 0 |
| 151 | 2 | .00 | 76 | 0 | 2 | 3 | 3 | 0 |
| 151 | 3 |  | 76 | 0 | 2 | 3 | 3 | 0 |
| 152 | 1 | 7.00 | 50 | 0 | 2 | 2 | 1 | 1 |
| 152 | 2 | 4.00 | 50 | 0 | 2 | 2 | 1 | 1 |
| 152 | 3 |  | 50 | 0 | 2 | 2 | 1 | 1 |
| 153 | 1 | 9.00 | 77 | 1 | 1 | 1 | 3 | 1 |
| 153 | 2 |  | 77 | 1 | 1 | 1 | 3 | 1 |
| 153 | 3 |  | 77 | 1 | 1 | 1 | 3 | 1 |
| 154 | 1 | 2.00 | 59 | 0 | 2 | 2 | 2 | 0 |
| 154 | 2 | .00 | 59 | 0 | 2 | 2 | 2 | 0 |
| 154 | 3 |  | 59 | 0 | 2 | 2 | 2 | 0 |
| 155 | 1 | 10.00 | 57 | 0 | 2 | 2 | 3 | 0 |
| 155 | 2 | 7.00 | 57 | 0 | 2 | 2 | 3 | 0 |
| 155 | 3 |  | 57 | 0 | 2 | 2 | 3 | 0 |
| 156 | 1 | 14.00 | 73 | 1 | 2 | 2 | 3 | 0 |
| 156 | 2 | .00 | 73 | 1 | 2 | 2 | 3 | 0 |
| 156 | 3 |  | 73 | 1 | 2 | 2 | 3 | 0 |
| 157 | 1 | 9.00 | 77 | 0 | 2 | 2 | 3 | 0 |
| 157 | 2 |  | 77 | 0 | 2 | 2 | 3 | 0 |
| 157 | 3 |  | 77 | 0 | 2 | 2 | 3 | 0 |
| 158 | 1 | 5.00 | 63 | 0 | 2 | 2 | 2 | 0 |
| 158 | 2 | 5.00 | 63 | 0 | 2 | 2 | 2 | 0 |
| 158 | 3 |  | 63 | 0 | 2 | 2 | 2 | 0 |
| 159 | 1 | .00 | 50 | 0 | 2 | 2 | 1 | 1 |
| 159 | 2 | 3.00 | 50 | 0 | 2 | 2 | 1 | 1 |
| 159 | 3 |  | 50 | 0 | 2 | 2 | 1 | 1 |
| 160 | 1 | 4.00 | 33 | 0 | 2 | 3 | 2 | 0 |
| 160 | 2 | .00 | 33 | 0 | 2 | 3 | 2 | 0 |
| 160 | 3 |  | 33 | 0 | 2 | 3 | 2 | 0 |
| 161 | 1 | 13.00 | 57 | 0 | 2 | 2 | 1 | 0 |
| 161 | 2 | 7.00 | 57 | 0 | 2 | 2 | 1 | 0 |
| 161 | 3 |  | 57 | 0 | 2 | 2 | 1 | 0 |
| 162 | 1 | 8.00 | 57 | 0 | 2 | 3 | 1 | 0 |
| 162 | 2 |  | 57 | 0 | 2 | 3 | 1 | 0 |
| 162 | 3 |  | 57 | 0 | 2 | 3 | 1 | 0 |
| 163 | 1 | 3.00 | 53 | 0 | 2 | 2 | 1 | 0 |
| 163 | 2 |  | 53 | 0 | 2 | 2 | 1 | 0 |
| 163 | 3 |  | 53 | 0 | 2 | 2 | 1 | 0 |
| 165 | 1 | 2.00 | 46 | 0 | 2 | 3 | 2 | 1 |
| 165 | 2 | 5.00 | 46 | 0 | 2 | 3 | 2 | 1 |
| 165 | 3 |  | 46 | 0 | 2 | 3 | 2 | 1 |
| 166 | 1 | 5.00 | 64 | 0 | 1 | 2 | 3 | 1 |
| 166 | 2 | 10.00 | 64 | 0 | 1 | 2 | 3 | 1 |
| 166 | 3 |  | 64 | 0 | 1 | 2 | 3 | 1 |
| 167 | 1 | 20.00 | 78 | 1 | 1 | 2 | 3 | 0 |
| 167 | 2 |  | 78 | 1 | 1 | 2 | 3 | 0 |
| 167 | 3 |  | 78 | 1 | 1 | 2 | 3 | 0 |
| 168 | 1 | .00 | 43 | 0 | 2 | 2 | 1 | 0 |
| 168 | 2 | .00 | 43 | 0 | 2 | 2 | 1 | 0 |
| 168 | 3 |  | 43 | 0 | 2 | 2 | 1 | 0 |
| 169 | 1 | 8.00 | 48 | 1 | 2 | 3 | 1 | 0 |
| 169 | 2 | 9.00 | 48 | 1 | 2 | 3 | 1 | 0 |
| 169 | 3 |  | 48 | 1 | 2 | 3 | 1 | 0 |
| 170 | 1 | .00 | 43 | 0 | 2 | 2 | 1 | 0 |
| 170 | 2 | 6.00 | 43 | 0 | 2 | 2 | 1 | 0 |
| 170 | 3 |  | 43 | 0 | 2 | 2 | 1 | 0 |
| 171 | 1 | 13.00 | 56 | 0 | 2 | 3 | 1 | 1 |
| 171 | 2 | 11.00 | 56 | 0 | 2 | 3 | 1 | 1 |
| 171 | 3 |  | 56 | 0 | 2 | 3 | 1 | 1 |
| 172 | 1 | 8.00 | 69 | 0 | 1 | 2 | 3 | 0 |
| 172 | 2 | 4.00 | 69 | 0 | 1 | 2 | 3 | 0 |
| 172 | 3 |  | 69 | 0 | 1 | 2 | 3 | 0 |
| 173 | 1 | 1.00 | 18 | 0 | 1 | 2 | 1 | 0 |
| 173 | 2 | 5.00 | 18 | 0 | 1 | 2 | 1 | 0 |
| 173 | 3 |  | 18 | 0 | 1 | 2 | 1 | 0 |
| 174 | 1 | 9.00 | 65 | 0 | 2 | 2 | 2 | 0 |
| 174 | 2 | 8.00 | 65 | 0 | 2 | 2 | 2 | 0 |
| 174 | 3 |  | 65 | 0 | 2 | 2 | 2 | 0 |
| 175 | 1 | 6.00 | 67 | 0 | 2 | 2 | 3 | 0 |
| 175 | 2 | .00 | 67 | 0 | 2 | 2 | 3 | 0 |
| 175 | 3 |  | 67 | 0 | 2 | 2 | 3 | 0 |
| 176 | 1 | 7.00 | 58 | 0 | 2 | 2 | 1 | 1 |
| 176 | 2 | 8.00 | 58 | 0 | 2 | 2 | 1 | 1 |
| 176 | 3 |  | 58 | 0 | 2 | 2 | 1 | 1 |
| 177 | 1 | 3.00 | 55 | 0 | 2 | 2 | 1 | 0 |
| 177 | 2 |  | 55 | 0 | 2 | 2 | 1 | 0 |
| 177 | 3 |  | 55 | 0 | 2 | 2 | 1 | 0 |
| 178 | 1 | 9.00 | 53 | 0 | 2 | 2 | 1 | 0 |
| 178 | 2 | .00 | 53 | 0 | 2 | 2 | 1 | 0 |
| 178 | 3 |  | 53 | 0 | 2 | 2 | 1 | 0 |
| 179 | 1 | 8.00 | 67 | 0 | 2 | 2 | 3 | 0 |
| 179 | 2 | .00 | 67 | 0 | 2 | 2 | 3 | 0 |
| 179 | 3 |  | 67 | 0 | 2 | 2 | 3 | 0 |
| 180 | 1 | 11.00 | 62 | 0 | 1 | 3 | 2 | 0 |
| 180 | 2 | 5.00 | 62 | 0 | 1 | 3 | 2 | 0 |
| 180 | 3 |  | 62 | 0 | 1 | 3 | 2 | 0 |
| 181 | 1 | 10.00 | 43 | 1 | 2 | 2 | 1 | 0 |
| 181 | 2 | 6.00 | 43 | 1 | 2 | 2 | 1 | 0 |
| 181 | 3 |  | 43 | 1 | 2 | 2 | 1 | 0 |
| 183 | 1 | .00 | 78 | 1 | 1 | 2 | 3 | 1 |
| 183 | 2 | 20.00 | 78 | 1 | 1 | 2 | 3 | 1 |
| 183 | 3 |  | 78 | 1 | 1 | 2 | 3 | 1 |
| 184 | 1 | 5.00 | 54 | 0 | 2 | 2 | 1 | 0 |
| 184 | 2 |  | 54 | 0 | 2 | 2 | 1 | 0 |
| 184 | 3 |  | 54 | 0 | 2 | 2 | 1 | 0 |
| 185 | 1 | .00 | 69 | 0 | 1 | 2 | 3 | 1 |
| 185 | 2 | 7.00 | 69 | 0 | 1 | 2 | 3 | 1 |
| 185 | 3 |  | 69 | 0 | 1 | 2 | 3 | 1 |
| 186 | 1 | .00 | 75 | 1 | 2 | 3 | 3 | 0 |
| 186 | 2 | 8.00 | 75 | 1 | 2 | 3 | 3 | 0 |
| 186 | 3 |  | 75 | 1 | 2 | 3 | 3 | 0 |
| 187 | 1 | .00 | 58 | 0 | 2 | 3 | 1 | 0 |
| 187 | 2 | 2.00 | 58 | 0 | 2 | 3 | 1 | 0 |
| 187 | 3 |  | 58 | 0 | 2 | 3 | 1 | 0 |
| 188 | 1 | 9.00 | 68 | 0 | 2 | 3 | 3 | 0 |
| 188 | 2 | 7.00 | 68 | 0 | 2 | 3 | 3 | 0 |
| 188 | 3 |  | 68 | 0 | 2 | 3 | 3 | 0 |
| 189 | 1 | 4.00 | 60 | 0 | 1 | 2 | 1 | 0 |
| 189 | 2 | .00 | 60 | 0 | 1 | 2 | 1 | 0 |
| 189 | 3 |  | 60 | 0 | 1 | 2 | 1 | 0 |
| 190 | 1 | .00 | 60 | 0 | 2 | 3 | 1 | 0 |
| 190 | 2 |  | 60 | 0 | 2 | 3 | 1 | 0 |
| 190 | 3 |  | 60 | 0 | 2 | 3 | 1 | 0 |
