# Supplementary material for: Sleep disturbance after acute coronary syndrome: A longitudinal study over 12 months
Source: PLoS One. 2022 Jun 3;17(6):e0269545. doi: 10.1371/journal.pone.0269545 (PMC9165780; doi:10.1371/journal.pone.0269545)
Supplement: S2 Dataset — (DOCX) [file pone.0269545.s002.docx]

| Participant number | Number of diseased coronary vessels (stenosis >50%) | Pain 0-10 | Fear of dying 0-10 | Helplessness 0-10 | Charlson comorbidity (category)  1=low risk  2= medium risk  3= high risk | History of depression  0=no  1=yes | history of sleep apnea  0=no  1=yes | sleeping pills  0=no  1=yes |
| --- | --- | --- | --- | --- | --- | --- | --- | --- |
| 2 | 1 | 10.0 | 8.0 | .0 | 1 | 0 | 0 | .00 |
| 2 | 1 | 10.0 | 8.0 | .0 | 1 | 0 | 0 | .00 |
| 2 | 1 | 10.0 | 8.0 | .0 | 1 | 0 | 0 | .00 |
| 3 | 1 | 8.0 | 4.0 | 6.0 | 2 | 0 | 0 | .00 |
| 3 | 1 | 8.0 | 4.0 | 6.0 | 2 | 0 | 0 | .00 |
| 3 | 1 | 8.0 | 4.0 | 6.0 | 2 | 0 | 0 | .00 |
| 4 | 2 | 7.0 | 5.0 | 8.0 | 1 | 1 | 0 | .00 |
| 4 | 2 | 7.0 | 5.0 | 8.0 | 1 | 1 | 0 | .00 |
| 4 | 2 | 7.0 | 5.0 | 8.0 | 1 | 1 | 0 |  |
| 5 | 2 | 8.0 | 3.0 | 7.0 | 1 | 0 | 0 | .00 |
| 5 | 2 | 8.0 | 3.0 | 7.0 | 1 | 0 | 0 | .00 |
| 5 | 2 | 8.0 | 3.0 | 7.0 | 1 | 0 | 0 | .00 |
| 6 | 2 | 8.0 | 7.0 | 5.0 | 3 | 0 | 0 | .00 |
| 6 | 2 | 8.0 | 7.0 | 5.0 | 3 | 0 | 0 | 1.00 |
| 6 | 2 | 8.0 | 7.0 | 5.0 | 3 | 0 | 0 | .00 |
| 7 | 2 | 8.0 | 5.0 | .0 | 1 | 0 | 0 | .00 |
| 7 | 2 | 8.0 | 5.0 | .0 | 1 | 0 | 0 | .00 |
| 7 | 2 | 8.0 | 5.0 | .0 | 1 | 0 | 0 | .00 |
| 8 | 3 | 5.0 | .0 | 7.0 | 2 | 0 | 0 | .00 |
| 8 | 3 | 5.0 | .0 | 7.0 | 2 | 0 | 0 | .00 |
| 8 | 3 | 5.0 | .0 | 7.0 | 2 | 0 | 0 | .00 |
| 9 | 2 | 9.0 | .0 | 10.0 | 2 | 0 | 0 | .00 |
| 9 | 2 | 9.0 | .0 | 10.0 | 2 | 0 | 0 | .00 |
| 9 | 2 | 9.0 | .0 | 10.0 | 2 | 0 | 0 | .00 |
| 10 | 1 | 10.0 | 4.0 | 10.0 | 1 | 0 | 0 | .00 |
| 10 | 1 | 10.0 | 4.0 | 10.0 | 1 | 0 | 0 | .00 |
| 10 | 1 | 10.0 | 4.0 | 10.0 | 1 | 0 | 0 | .00 |
| 11 | 2 | 9.0 | 8.0 | 5.0 | 1 | 0 | 0 | .00 |
| 11 | 2 | 9.0 | 8.0 | 5.0 | 1 | 0 | 0 | .00 |
| 11 | 2 | 9.0 | 8.0 | 5.0 | 1 | 0 | 0 | .00 |
| 12 | 1 | 9.5 | 9.0 | 9.0 | 1 | 0 | 1 | .00 |
| 12 | 1 | 9.5 | 9.0 | 9.0 | 1 | 0 | 1 |  |
| 12 | 1 | 9.5 | 9.0 | 9.0 | 1 | 0 | 1 |  |
| 13 | 3 | 7.0 | 8.0 | 3.0 | 1 | 0 | 0 | .00 |
| 13 | 3 | 7.0 | 8.0 | 3.0 | 1 | 0 | 0 |  |
| 13 | 3 | 7.0 | 8.0 | 3.0 | 1 | 0 | 0 |  |
| 14 | 1 | 10.0 | .0 | 6.0 | 1 | 0 | 0 | .00 |
| 14 | 1 | 10.0 | .0 | 6.0 | 1 | 0 | 0 | .00 |
| 14 | 1 | 10.0 | .0 | 6.0 | 1 | 0 | 0 | .00 |
| 16 | 1 | 10.0 | .0 | 5.0 | 1 | 0 | 0 | .00 |
| 16 | 1 | 10.0 | .0 | 5.0 | 1 | 0 | 0 |  |
| 16 | 1 | 10.0 | .0 | 5.0 | 1 | 0 | 0 |  |
| 17 | 3 | 5.0 | 6.0 | 6.0 | 1 | 0 | 0 | .00 |
| 17 | 3 | 5.0 | 6.0 | 6.0 | 1 | 0 | 0 | .00 |
| 17 | 3 | 5.0 | 6.0 | 6.0 | 1 | 0 | 0 | .00 |
| 18 | 3 | 10.0 | 10.0 | 8.0 | 3 | 0 | 0 | .00 |
| 18 | 3 | 10.0 | 10.0 | 8.0 | 3 | 0 | 0 |  |
| 18 | 3 | 10.0 | 10.0 | 8.0 | 3 | 0 | 0 |  |
| 19 | 1 | 5.0 | 7.0 | 5.0 | 1 | 0 | 0 | .00 |
| 19 | 1 | 5.0 | 7.0 | 5.0 | 1 | 0 | 0 | .00 |
| 19 | 1 | 5.0 | 7.0 | 5.0 | 1 | 0 | 0 | .00 |
| 21 | 3 | 8.0 | 4.0 | 10.0 | 2 | 1 | 1 | .00 |
| 21 | 3 | 8.0 | 4.0 | 10.0 | 2 | 1 | 1 | 1.00 |
| 21 | 3 | 8.0 | 4.0 | 10.0 | 2 | 1 | 1 | .00 |
| 22 | 2 | 9.0 | 2.0 | 7.0 | 1 | 0 | 0 | .00 |
| 22 | 2 | 9.0 | 2.0 | 7.0 | 1 | 0 | 0 | .00 |
| 22 | 2 | 9.0 | 2.0 | 7.0 | 1 | 0 | 0 | .00 |
| 24 | 1 | 8.0 | 8.0 | 3.0 | 1 | 0 | 0 | .00 |
| 24 | 1 | 8.0 | 8.0 | 3.0 | 1 | 0 | 0 | .00 |
| 24 | 1 | 8.0 | 8.0 | 3.0 | 1 | 0 | 0 | .00 |
| 25 | 1 | 10.0 | .0 | 7.0 | 2 | 0 | 1 | .00 |
| 25 | 1 | 10.0 | .0 | 7.0 | 2 | 0 | 1 | .00 |
| 25 | 1 | 10.0 | .0 | 7.0 | 2 | 0 | 1 | .00 |
| 26 | 3 | 8.0 | 5.0 | 8.0 | 3 | 0 | 0 | .00 |
| 26 | 3 | 8.0 | 5.0 | 8.0 | 3 | 0 | 0 | .00 |
| 26 | 3 | 8.0 | 5.0 | 8.0 | 3 | 0 | 0 | .00 |
| 27 | 3 | 7.0 | 10.0 | 10.0 | 1 | 1 | 0 | .00 |
| 27 | 3 | 7.0 | 10.0 | 10.0 | 1 | 1 | 0 |  |
| 27 | 3 | 7.0 | 10.0 | 10.0 | 1 | 1 | 0 |  |
| 28 | 2 | 7.0 | 6.0 | 3.0 | 3 | 0 | 0 | .00 |
| 28 | 2 | 7.0 | 6.0 | 3.0 | 3 | 0 | 0 | .00 |
| 28 | 2 | 7.0 | 6.0 | 3.0 | 3 | 0 | 0 | .00 |
| 29 | 1 | 8.0 | 4.0 | 7.0 | 1 | 0 | 0 | .00 |
| 29 | 1 | 8.0 | 4.0 | 7.0 | 1 | 0 | 0 | .00 |
| 29 | 1 | 8.0 | 4.0 | 7.0 | 1 | 0 | 0 | .00 |
| 30 | 3 | 9.0 | 9.0 | 2.0 | 3 | 1 | 1 | .00 |
| 30 | 3 | 9.0 | 9.0 | 2.0 | 3 | 1 | 1 | .00 |
| 30 | 3 | 9.0 | 9.0 | 2.0 | 3 | 1 | 1 | .00 |
| 31 | 3 | 10.0 | .0 | 8.0 | 1 | 0 | 0 | .00 |
| 31 | 3 | 10.0 | .0 | 8.0 | 1 | 0 | 0 | .00 |
| 31 | 3 | 10.0 | .0 | 8.0 | 1 | 0 | 0 | .00 |
| 32 | 3 | 8.0 | 7.0 | 7.0 | 1 | 0 | 0 | .00 |
| 32 | 3 | 8.0 | 7.0 | 7.0 | 1 | 0 | 0 | .00 |
| 32 | 3 | 8.0 | 7.0 | 7.0 | 1 | 0 | 0 | .00 |
| 33 | 3 | 10.0 | 9.0 | .0 | 2 | 0 | 0 | .00 |
| 33 | 3 | 10.0 | 9.0 | .0 | 2 | 0 | 0 |  |
| 33 | 3 | 10.0 | 9.0 | .0 | 2 | 0 | 0 |  |
| 34 | 2 | 10.0 | 7.0 | 3.0 | 2 | 1 | 0 | 1.00 |
| 34 | 2 | 10.0 | 7.0 | 3.0 | 2 | 1 | 0 | .00 |
| 34 | 2 | 10.0 | 7.0 | 3.0 | 2 | 1 | 0 | .00 |
| 35 | 1 | 6.0 | 10.0 | 8.0 | 1 | 0 | 0 | .00 |
| 35 | 1 | 6.0 | 10.0 | 8.0 | 1 | 0 | 0 | .00 |
| 35 | 1 | 6.0 | 10.0 | 8.0 | 1 | 0 | 0 |  |
| 36 | 2 | 7.0 | 7.0 | 3.0 | 3 | 0 | 0 | .00 |
| 36 | 2 | 7.0 | 7.0 | 3.0 | 3 | 0 | 0 |  |
| 36 | 2 | 7.0 | 7.0 | 3.0 | 3 | 0 | 0 |  |
| 37 | 3 | 10.0 | 6.0 | 5.0 | 2 | 0 | 0 | .00 |
| 37 | 3 | 10.0 | 6.0 | 5.0 | 2 | 0 | 0 |  |
| 37 | 3 | 10.0 | 6.0 | 5.0 | 2 | 0 | 0 |  |
| 38 | 3 | 9.0 | 10.0 | 5.0 | 3 | 0 | 0 | .00 |
| 38 | 3 | 9.0 | 10.0 | 5.0 | 3 | 0 | 0 | .00 |
| 38 | 3 | 9.0 | 10.0 | 5.0 | 3 | 0 | 0 | .00 |
| 39 | 2 | 8.0 | 8.0 | .0 | 1 | 1 | 0 | .00 |
| 39 | 2 | 8.0 | 8.0 | .0 | 1 | 1 | 0 | .00 |
| 39 | 2 | 8.0 | 8.0 | .0 | 1 | 1 | 0 | .00 |
| 40 | 1 | 9.0 | 6.0 | 6.0 | 3 | 0 | 0 | .00 |
| 40 | 1 | 9.0 | 6.0 | 6.0 | 3 | 0 | 0 | .00 |
| 40 | 1 | 9.0 | 6.0 | 6.0 | 3 | 0 | 0 | .00 |
| 41 | 2 | 7.0 | .0 | 8.0 | 3 | 0 | 0 | .00 |
| 41 | 2 | 7.0 | .0 | 8.0 | 3 | 0 | 0 | .00 |
| 41 | 2 | 7.0 | .0 | 8.0 | 3 | 0 | 0 | .00 |
| 42 | 1 | 8.0 | 8.0 | 8.0 | 1 | 1 | 0 | .00 |
| 42 | 1 | 8.0 | 8.0 | 8.0 | 1 | 1 | 0 | .00 |
| 42 | 1 | 8.0 | 8.0 | 8.0 | 1 | 1 | 0 | .00 |
| 43 | 1 | 8.0 | 5.0 | 4.0 | 1 | 0 | 1 | .00 |
| 43 | 1 | 8.0 | 5.0 | 4.0 | 1 | 0 | 1 | .00 |
| 43 | 1 | 8.0 | 5.0 | 4.0 | 1 | 0 | 1 | .00 |
| 44 | 1 | 5.0 | 5.0 | 7.0 | 1 | 0 | 1 | .00 |
| 44 | 1 | 5.0 | 5.0 | 7.0 | 1 | 0 | 1 | .00 |
| 44 | 1 | 5.0 | 5.0 | 7.0 | 1 | 0 | 1 | .00 |
| 45 | 3 | 6.0 | 5.0 | 4.0 | 1 | 0 | 1 | .00 |
| 45 | 3 | 6.0 | 5.0 | 4.0 | 1 | 0 | 1 | .00 |
| 45 | 3 | 6.0 | 5.0 | 4.0 | 1 | 0 | 1 | .00 |
| 46 | 1 | 7.0 | 7.0 | 7.0 | 1 | 0 | 0 | .00 |
| 46 | 1 | 7.0 | 7.0 | 7.0 | 1 | 0 | 0 | .00 |
| 46 | 1 | 7.0 | 7.0 | 7.0 | 1 | 0 | 0 | .00 |
| 47 | 3 | 9.0 | 10.0 | 5.0 | 1 | 1 | 0 | .00 |
| 47 | 3 | 9.0 | 10.0 | 5.0 | 1 | 1 | 0 | .00 |
| 47 | 3 | 9.0 | 10.0 | 5.0 | 1 | 1 | 0 |  |
| 48 | 3 | 7.0 | 3.0 | 7.0 | 1 | 0 | 1 | .00 |
| 48 | 3 | 7.0 | 3.0 | 7.0 | 1 | 0 | 1 | .00 |
| 48 | 3 | 7.0 | 3.0 | 7.0 | 1 | 0 | 1 | .00 |
| 49 | 1 | 8.0 | 6.0 | 8.0 | 1 | 0 | 0 | .00 |
| 49 | 1 | 8.0 | 6.0 | 8.0 | 1 | 0 | 0 | .00 |
| 49 | 1 | 8.0 | 6.0 | 8.0 | 1 | 0 | 0 | .00 |
| 50 | 1 | 10.0 | 8.0 | 10.0 | 1 | 0 | 0 | .00 |
| 50 | 1 | 10.0 | 8.0 | 10.0 | 1 | 0 | 0 | .00 |
| 50 | 1 | 10.0 | 8.0 | 10.0 | 1 | 0 | 0 | .00 |
| 51 | 1 | 8.0 | 8.0 | 5.0 | 2 | 1 | 0 | .00 |
| 51 | 1 | 8.0 | 8.0 | 5.0 | 2 | 1 | 0 | 1.00 |
| 51 | 1 | 8.0 | 8.0 | 5.0 | 2 | 1 | 0 | 1.00 |
| 52 | 3 | 6.0 | 4.0 | 7.0 | 3 | 0 | 0 | 1.00 |
| 52 | 3 | 6.0 | 4.0 | 7.0 | 3 | 0 | 0 |  |
| 52 | 3 | 6.0 | 4.0 | 7.0 | 3 | 0 | 0 |  |
| 53 | 1 | 5.0 | 5.0 | 3.0 | 1 | 0 | 0 | .00 |
| 53 | 1 | 5.0 | 5.0 | 3.0 | 1 | 0 | 0 | .00 |
| 53 | 1 | 5.0 | 5.0 | 3.0 | 1 | 0 | 0 | 1.00 |
| 54 | 2 | 6.0 | 4.0 | 8.0 | 3 | 1 | 0 | .00 |
| 54 | 2 | 6.0 | 4.0 | 8.0 | 3 | 1 | 0 | .00 |
| 54 | 2 | 6.0 | 4.0 | 8.0 | 3 | 1 | 0 | .00 |
| 55 | 1 | 8.0 | 1.0 | 5.0 | 2 | 1 | 0 | .00 |
| 55 | 1 | 8.0 | 1.0 | 5.0 | 2 | 1 | 0 | .00 |
| 55 | 1 | 8.0 | 1.0 | 5.0 | 2 | 1 | 0 | .00 |
| 56 | 1 | 8.0 | 8.0 | 2.0 | 3 | 1 | 0 | .00 |
| 56 | 1 | 8.0 | 8.0 | 2.0 | 3 | 1 | 0 | 1.00 |
| 56 | 1 | 8.0 | 8.0 | 2.0 | 3 | 1 | 0 | 1.00 |
| 57 | 2 | 10.0 | 8.0 | .0 | 1 | 0 | 0 | .00 |
| 57 | 2 | 10.0 | 8.0 | .0 | 1 | 0 | 0 | .00 |
| 57 | 2 | 10.0 | 8.0 | .0 | 1 | 0 | 0 | .00 |
| 58 | 3 | 10.0 | 8.0 | 7.0 | 3 | 0 | 0 | .00 |
| 58 | 3 | 10.0 | 8.0 | 7.0 | 3 | 0 | 0 | .00 |
| 58 | 3 | 10.0 | 8.0 | 7.0 | 3 | 0 | 0 | .00 |
| 59 | 2 | 9.0 | 8.0 | 9.0 | 1 | 0 | 0 | .00 |
| 59 | 2 | 9.0 | 8.0 | 9.0 | 1 | 0 | 0 | .00 |
| 59 | 2 | 9.0 | 8.0 | 9.0 | 1 | 0 | 0 | .00 |
| 60 | 3 | 10.0 | 2.0 | 5.0 | 2 | 0 | 0 | .00 |
| 60 | 3 | 10.0 | 2.0 | 5.0 | 2 | 0 | 0 | .00 |
| 60 | 3 | 10.0 | 2.0 | 5.0 | 2 | 0 | 0 | .00 |
| 61 | 2 | 7.0 | 6.0 | 2.0 | 3 | 0 | 0 | .00 |
| 61 | 2 | 7.0 | 6.0 | 2.0 | 3 | 0 | 0 |  |
| 61 | 2 | 7.0 | 6.0 | 2.0 | 3 | 0 | 0 |  |
| 62 | 2 | 9.0 | 4.0 | 9.0 | 1 | 0 | 0 | .00 |
| 62 | 2 | 9.0 | 4.0 | 9.0 | 1 | 0 | 0 |  |
| 62 | 2 | 9.0 | 4.0 | 9.0 | 1 | 0 | 0 |  |
| 63 | 2 | 7.0 | 8.0 | 9.0 | 1 | 1 | 0 | .00 |
| 63 | 2 | 7.0 | 8.0 | 9.0 | 1 | 1 | 0 | .00 |
| 63 | 2 | 7.0 | 8.0 | 9.0 | 1 | 1 | 0 |  |
| 64 | 1 | 10.0 | 2.0 | 5.0 | 1 | 0 | 0 | .00 |
| 64 | 1 | 10.0 | 2.0 | 5.0 | 1 | 0 | 0 | .00 |
| 64 | 1 | 10.0 | 2.0 | 5.0 | 1 | 0 | 0 | .00 |
| 65 | 2 | 9.0 | 2.0 | 5.0 | 2 | 0 | 0 | .00 |
| 65 | 2 | 9.0 | 2.0 | 5.0 | 2 | 0 | 0 | .00 |
| 65 | 2 | 9.0 | 2.0 | 5.0 | 2 | 0 | 0 | 1.00 |
| 66 | 1 | 9.0 | 5.0 | 4.0 | 3 | 0 | 0 | .00 |
| 66 | 1 | 9.0 | 5.0 | 4.0 | 3 | 0 | 0 | .00 |
| 66 | 1 | 9.0 | 5.0 | 4.0 | 3 | 0 | 0 | 1.00 |
| 67 | 3 | 10.0 | 10.0 | 10.0 | 2 | 0 | 0 | 1.00 |
| 67 | 3 | 10.0 | 10.0 | 10.0 | 2 | 0 | 0 | .00 |
| 67 | 3 | 10.0 | 10.0 | 10.0 | 2 | 0 | 0 | .00 |
| 68 | 3 | 6.0 | 6.0 | 5.0 | 1 | 0 | 0 | .00 |
| 68 | 3 | 6.0 | 6.0 | 5.0 | 1 | 0 | 0 | .00 |
| 68 | 3 | 6.0 | 6.0 | 5.0 | 1 | 0 | 0 | .00 |
| 69 | 1 | 10.0 | 10.0 | 10.0 | 1 | 0 | 0 | .00 |
| 69 | 1 | 10.0 | 10.0 | 10.0 | 1 | 0 | 0 | .00 |
| 69 | 1 | 10.0 | 10.0 | 10.0 | 1 | 0 | 0 |  |
| 72 | 2 | 8.0 | 5.0 | 2.0 | 3 | 0 | 0 | .00 |
| 72 | 2 | 8.0 | 5.0 | 2.0 | 3 | 0 | 0 | .00 |
| 72 | 2 | 8.0 | 5.0 | 2.0 | 3 | 0 | 0 | .00 |
| 73 | 3 | 8.0 | 9.0 | .0 | 3 | 0 | 0 | .00 |
| 73 | 3 | 8.0 | 9.0 | .0 | 3 | 0 | 0 | .00 |
| 73 | 3 | 8.0 | 9.0 | .0 | 3 | 0 | 0 | 1.00 |
| 74 | 1 | 7.0 | 5.0 | .0 | 2 | 0 | 0 | .00 |
| 74 | 1 | 7.0 | 5.0 | .0 | 2 | 0 | 0 |  |
| 74 | 1 | 7.0 | 5.0 | .0 | 2 | 0 | 0 |  |
| 75 | 2 | 8.0 | 3.0 | 6.0 | 3 | 1 | 0 | .00 |
| 75 | 2 | 8.0 | 3.0 | 6.0 | 3 | 1 | 0 |  |
| 75 | 2 | 8.0 | 3.0 | 6.0 | 3 | 1 | 0 |  |
| 76 | 3 | 5.0 | .0 | 5.0 | 2 | 0 | 0 | .00 |
| 76 | 3 | 5.0 | .0 | 5.0 | 2 | 0 | 0 |  |
| 76 | 3 | 5.0 | .0 | 5.0 | 2 | 0 | 0 | .00 |
| 77 | 1 | 6.0 | 5.0 | 2.0 | 1 | 0 | 0 | .00 |
| 77 | 1 | 6.0 | 5.0 | 2.0 | 1 | 0 | 0 |  |
| 77 | 1 | 6.0 | 5.0 | 2.0 | 1 | 0 | 0 |  |
| 78 | 3 | 8.0 | 7.0 | 9.0 | 2 | 1 | 1 | .00 |
| 78 | 3 | 8.0 | 7.0 | 9.0 | 2 | 1 | 1 | .00 |
| 78 | 3 | 8.0 | 7.0 | 9.0 | 2 | 1 | 1 | 1.00 |
| 80 | 3 | 9.0 | 9.0 | 10.0 | 3 | 1 | 0 | 1.00 |
| 80 | 3 | 9.0 | 9.0 | 10.0 | 3 | 1 | 0 |  |
| 80 | 3 | 9.0 | 9.0 | 10.0 | 3 | 1 | 0 |  |
| 81 | 2 | 9.0 | 9.0 | 4.0 | 1 | 0 | 0 | .00 |
| 81 | 2 | 9.0 | 9.0 | 4.0 | 1 | 0 | 0 | .00 |
| 81 | 2 | 9.0 | 9.0 | 4.0 | 1 | 0 | 0 | .00 |
| 82 | 2 | 5.0 | 7.0 | 2.0 | 2 | 0 | 0 | .00 |
| 82 | 2 | 5.0 | 7.0 | 2.0 | 2 | 0 | 0 |  |
| 82 | 2 | 5.0 | 7.0 | 2.0 | 2 | 0 | 0 | .00 |
| 83 | 2 | 8.0 | 5.0 | 6.0 | 2 | 1 | 1 | .00 |
| 83 | 2 | 8.0 | 5.0 | 6.0 | 2 | 1 | 1 | .00 |
| 83 | 2 | 8.0 | 5.0 | 6.0 | 2 | 1 | 1 | .00 |
| 84 | 2 | 8.0 | .0 | 5.0 | 2 | 0 | 0 | .00 |
| 84 | 2 | 8.0 | .0 | 5.0 | 2 | 0 | 0 | .00 |
| 84 | 2 | 8.0 | .0 | 5.0 | 2 | 0 | 0 | .00 |
| 85 | 2 | 9.5 | 3.0 | 8.0 | 3 | 0 | 0 | .00 |
| 85 | 2 | 9.5 | 3.0 | 8.0 | 3 | 0 | 0 | .00 |
| 85 | 2 | 9.5 | 3.0 | 8.0 | 3 | 0 | 0 | .00 |
| 86 | 3 | 8.0 | 5.0 | 8.0 | 1 | 0 | 0 | .00 |
| 86 | 3 | 8.0 | 5.0 | 8.0 | 1 | 0 | 0 | .00 |
| 86 | 3 | 8.0 | 5.0 | 8.0 | 1 | 0 | 0 | .00 |
| 87 | 1 | 5.0 | 5.0 | .0 | 2 | 1 | 0 | .00 |
| 87 | 1 | 5.0 | 5.0 | .0 | 2 | 1 | 0 | .00 |
| 87 | 1 | 5.0 | 5.0 | .0 | 2 | 1 | 0 | .00 |
| 88 | 3 | 10.0 | 6.0 | 7.0 | 1 | 0 | 0 | .00 |
| 88 | 3 | 10.0 | 6.0 | 7.0 | 1 | 0 | 0 | .00 |
| 88 | 3 | 10.0 | 6.0 | 7.0 | 1 | 0 | 0 | .00 |
| 90 | 0 | 5.0 | 8.0 | 5.0 | 2 | 0 | 0 | 1.00 |
| 90 | 0 | 5.0 | 8.0 | 5.0 | 2 | 0 | 0 | .00 |
| 90 | 0 | 5.0 | 8.0 | 5.0 | 2 | 0 | 0 | .00 |
| 91 | 1 | 5.0 | 10.0 | 5.0 | 1 | 0 | 0 | .00 |
| 91 | 1 | 5.0 | 10.0 | 5.0 | 1 | 0 | 0 | .00 |
| 91 | 1 | 5.0 | 10.0 | 5.0 | 1 | 0 | 0 | .00 |
| 92 | 1 | 5.0 | 7.0 | 7.0 | 1 | 0 | 0 | .00 |
| 92 | 1 | 5.0 | 7.0 | 7.0 | 1 | 0 | 0 | .00 |
| 92 | 1 | 5.0 | 7.0 | 7.0 | 1 | 0 | 0 | .00 |
| 93 | 2 | 6.0 | 6.0 | 2.0 | 1 | 0 | 0 | .00 |
| 93 | 2 | 6.0 | 6.0 | 2.0 | 1 | 0 | 0 | .00 |
| 93 | 2 | 6.0 | 6.0 | 2.0 | 1 | 0 | 0 | .00 |
| 94 | 1 | 5.0 | 2.0 | 6.0 | 1 | 0 | 0 | .00 |
| 94 | 1 | 5.0 | 2.0 | 6.0 | 1 | 0 | 0 | .00 |
| 94 | 1 | 5.0 | 2.0 | 6.0 | 1 | 0 | 0 | .00 |
| 95 | 2 | 6.0 | 4.0 | 8.0 | 2 | 0 | 0 | .00 |
| 95 | 2 | 6.0 | 4.0 | 8.0 | 2 | 0 | 0 | .00 |
| 95 | 2 | 6.0 | 4.0 | 8.0 | 2 | 0 | 0 |  |
| 96 | 1 | 9.0 | 8.0 | 8.0 | 1 | 1 | 0 | 1.00 |
| 96 | 1 | 9.0 | 8.0 | 8.0 | 1 | 1 | 0 |  |
| 96 | 1 | 9.0 | 8.0 | 8.0 | 1 | 1 | 0 |  |
| 97 | 1 | 10.0 | 2.0 | 3.0 | 3 | 0 | 0 | .00 |
| 97 | 1 | 10.0 | 2.0 | 3.0 | 3 | 0 | 0 | .00 |
| 97 | 1 | 10.0 | 2.0 | 3.0 | 3 | 0 | 0 | .00 |
| 98 | 1 | 8.0 | 6.0 | 8.0 | 1 | 0 | 0 | .00 |
| 98 | 1 | 8.0 | 6.0 | 8.0 | 1 | 0 | 0 | 1.00 |
| 98 | 1 | 8.0 | 6.0 | 8.0 | 1 | 0 | 0 | 1.00 |
| 99 | 3 | 9.0 | 5.0 | 8.0 | 1 | 0 | 0 | .00 |
| 99 | 3 | 9.0 | 5.0 | 8.0 | 1 | 0 | 0 | .00 |
| 99 | 3 | 9.0 | 5.0 | 8.0 | 1 | 0 | 0 | .00 |
| 100 | 1 | 7.0 | 2.0 | 5.0 | 2 | 0 | 0 | .00 |
| 100 | 1 | 7.0 | 2.0 | 5.0 | 2 | 0 | 0 | .00 |
| 100 | 1 | 7.0 | 2.0 | 5.0 | 2 | 0 | 0 | .00 |
| 101 | 3 | 6.0 | 4.0 | 5.0 | 3 | 0 | 0 | .00 |
| 101 | 3 | 6.0 | 4.0 | 5.0 | 3 | 0 | 0 | .00 |
| 101 | 3 | 6.0 | 4.0 | 5.0 | 3 | 0 | 0 | .00 |
| 102 | 2 | 10.0 | 5.0 | 10.0 | 1 | 1 | 0 | .00 |
| 102 | 2 | 10.0 | 5.0 | 10.0 | 1 | 1 | 0 | .00 |
| 102 | 2 | 10.0 | 5.0 | 10.0 | 1 | 1 | 0 | .00 |
| 103 | 3 | 10.0 | 5.0 | 5.0 | 2 | 0 | 0 | .00 |
| 103 | 3 | 10.0 | 5.0 | 5.0 | 2 | 0 | 0 |  |
| 103 | 3 | 10.0 | 5.0 | 5.0 | 2 | 0 | 0 |  |
| 104 | 2 | 10.0 | 8.0 | 2.0 | 3 | 1 | 0 | .00 |
| 104 | 2 | 10.0 | 8.0 | 2.0 | 3 | 1 | 0 | .00 |
| 104 | 2 | 10.0 | 8.0 | 2.0 | 3 | 1 | 0 | .00 |
| 105 | 2 | 6.0 | 4.0 | 2.0 | 2 | 0 | 0 | .00 |
| 105 | 2 | 6.0 | 4.0 | 2.0 | 2 | 0 | 0 |  |
| 105 | 2 | 6.0 | 4.0 | 2.0 | 2 | 0 | 0 |  |
| 106 | 1 | 6.0 | 4.0 | 4.0 | 2 | 0 | 0 | .00 |
| 106 | 1 | 6.0 | 4.0 | 4.0 | 2 | 0 | 0 | .00 |
| 106 | 1 | 6.0 | 4.0 | 4.0 | 2 | 0 | 0 | .00 |
| 107 | 2 | 9.0 | 8.0 | 6.0 | 1 | 1 | 0 | .00 |
| 107 | 2 | 9.0 | 8.0 | 6.0 | 1 | 1 | 0 | .00 |
| 107 | 2 | 9.0 | 8.0 | 6.0 | 1 | 1 | 0 | .00 |
| 108 | 2 | 10.0 | 7.0 | 7.0 | 1 | 1 | 0 | .00 |
| 108 | 2 | 10.0 | 7.0 | 7.0 | 1 | 1 | 0 | .00 |
| 108 | 2 | 10.0 | 7.0 | 7.0 | 1 | 1 | 0 | .00 |
| 109 | 3 | 9.0 | 7.0 | 8.0 | 1 | 0 | 0 | .00 |
| 109 | 3 | 9.0 | 7.0 | 8.0 | 1 | 0 | 0 | .00 |
| 109 | 3 | 9.0 | 7.0 | 8.0 | 1 | 0 | 0 | .00 |
| 110 | 2 | 10.0 | 3.0 | 5.0 | 3 | 0 | 0 | 1.00 |
| 110 | 2 | 10.0 | 3.0 | 5.0 | 3 | 0 | 0 |  |
| 110 | 2 | 10.0 | 3.0 | 5.0 | 3 | 0 | 0 |  |
| 111 | 1 | 10.0 | 4.0 | 7.0 | 1 | 0 | 1 | .00 |
| 111 | 1 | 10.0 | 4.0 | 7.0 | 1 | 0 | 1 | .00 |
| 111 | 1 | 10.0 | 4.0 | 7.0 | 1 | 0 | 1 | .00 |
| 112 | 3 | 9.0 | .0 | 8.0 | 1 | 0 | 1 | .00 |
| 112 | 3 | 9.0 | .0 | 8.0 | 1 | 0 | 1 | .00 |
| 112 | 3 | 9.0 | .0 | 8.0 | 1 | 0 | 1 |  |
| 113 | 3 | 9.0 | .0 | 2.0 | 1 | 1 | 0 | .00 |
| 113 | 3 | 9.0 | .0 | 2.0 | 1 | 1 | 0 | .00 |
| 113 | 3 | 9.0 | .0 | 2.0 | 1 | 1 | 0 | .00 |
| 114 | 3 | 8.0 | 8.0 | 6.0 | 1 | 0 | 0 | .00 |
| 114 | 3 | 8.0 | 8.0 | 6.0 | 1 | 0 | 0 | .00 |
| 114 | 3 | 8.0 | 8.0 | 6.0 | 1 | 0 | 0 | .00 |
| 115 | 1 | 7.0 | 5.0 | 7.0 | 1 | 0 | 0 | .00 |
| 115 | 1 | 7.0 | 5.0 | 7.0 | 1 | 0 | 0 | .00 |
| 115 | 1 | 7.0 | 5.0 | 7.0 | 1 | 0 | 0 | .00 |
| 116 | 1 | 8.0 | 5.0 | 7.0 | 1 | 1 | 0 | .00 |
| 116 | 1 | 8.0 | 5.0 | 7.0 | 1 | 1 | 0 | 1.00 |
| 116 | 1 | 8.0 | 5.0 | 7.0 | 1 | 1 | 0 | .00 |
| 117 | 3 | 8.0 | 8.0 | 3.0 | 3 | 1 | 0 | .00 |
| 117 | 3 | 8.0 | 8.0 | 3.0 | 3 | 1 | 0 | .00 |
| 117 | 3 | 8.0 | 8.0 | 3.0 | 3 | 1 | 0 | .00 |
| 118 | 3 | 9.0 | 9.0 | 6.0 | 3 | 0 | 0 | .00 |
| 118 | 3 | 9.0 | 9.0 | 6.0 | 3 | 0 | 0 |  |
| 118 | 3 | 9.0 | 9.0 | 6.0 | 3 | 0 | 0 |  |
| 119 | 1 | 5.0 | 5.0 | 5.0 | 1 | 0 | 0 | .00 |
| 119 | 1 | 5.0 | 5.0 | 5.0 | 1 | 0 | 0 | .00 |
| 119 | 1 | 5.0 | 5.0 | 5.0 | 1 | 0 | 0 | .00 |
| 120 | 2 | 5.0 | .0 | 5.0 | 3 | 1 | 0 | .00 |
| 120 | 2 | 5.0 | .0 | 5.0 | 3 | 1 | 0 | .00 |
| 120 | 2 | 5.0 | .0 | 5.0 | 3 | 1 | 0 | .00 |
| 121 | 0 | 6.0 | 5.0 | 4.0 | 2 | 0 | 0 | .00 |
| 121 | 0 | 6.0 | 5.0 | 4.0 | 2 | 0 | 0 | .00 |
| 121 | 0 | 6.0 | 5.0 | 4.0 | 2 | 0 | 0 | .00 |
| 122 | 1 | 10.0 | 3.0 | 7.0 | 1 | 0 | 0 | .00 |
| 122 | 1 | 10.0 | 3.0 | 7.0 | 1 | 0 | 0 |  |
| 122 | 1 | 10.0 | 3.0 | 7.0 | 1 | 0 | 0 |  |
| 123 | 1 | 10.0 | 8.0 | 2.0 | 1 | 0 | 0 | .00 |
| 123 | 1 | 10.0 | 8.0 | 2.0 | 1 | 0 | 0 | .00 |
| 123 | 1 | 10.0 | 8.0 | 2.0 | 1 | 0 | 0 | .00 |
| 124 | 1 | 9.0 | 8.0 | 1.0 | 1 | 0 | 0 | .00 |
| 124 | 1 | 9.0 | 8.0 | 1.0 | 1 | 0 | 0 |  |
| 124 | 1 | 9.0 | 8.0 | 1.0 | 1 | 0 | 0 |  |
| 125 | 1 | 6.0 | 8.0 | 7.0 | 2 | 0 | 0 | .00 |
| 125 | 1 | 6.0 | 8.0 | 7.0 | 2 | 0 | 0 | .00 |
| 125 | 1 | 6.0 | 8.0 | 7.0 | 2 | 0 | 0 | .00 |
| 126 | 2 | 8.0 | 3.0 | 5.0 | 1 | 0 | 0 | .00 |
| 126 | 2 | 8.0 | 3.0 | 5.0 | 1 | 0 | 0 | .00 |
| 126 | 2 | 8.0 | 3.0 | 5.0 | 1 | 0 | 0 | .00 |
| 127 | 2 | 10.0 | 10.0 | 7.0 | 2 | 0 | 0 | .00 |
| 127 | 2 | 10.0 | 10.0 | 7.0 | 2 | 0 | 0 | .00 |
| 127 | 2 | 10.0 | 10.0 | 7.0 | 2 | 0 | 0 | .00 |
| 128 | 3 | 8.0 | 7.0 | 3.0 | 1 | 0 | 0 | .00 |
| 128 | 3 | 8.0 | 7.0 | 3.0 | 1 | 0 | 0 | .00 |
| 128 | 3 | 8.0 | 7.0 | 3.0 | 1 | 0 | 0 | .00 |
| 129 | 2 | 9.0 | .0 | 10.0 | 3 | 1 | 0 | .00 |
| 129 | 2 | 9.0 | .0 | 10.0 | 3 | 1 | 0 | 1.00 |
| 129 | 2 | 9.0 | .0 | 10.0 | 3 | 1 | 0 | 1.00 |
| 130 | 1 | 10.0 | 7.0 | 6.0 | 3 | 1 | 0 | .00 |
| 130 | 1 | 10.0 | 7.0 | 6.0 | 3 | 1 | 0 | .00 |
| 130 | 1 | 10.0 | 7.0 | 6.0 | 3 | 1 | 0 | .00 |
| 131 | 1 | 7.0 | 8.0 | 10.0 | 2 | 0 | 0 | .00 |
| 131 | 1 | 7.0 | 8.0 | 10.0 | 2 | 0 | 0 | .00 |
| 131 | 1 | 7.0 | 8.0 | 10.0 | 2 | 0 | 0 | .00 |
| 132 | 1 | 9.0 | 5.0 | .0 | 1 | 0 | 0 | .00 |
| 132 | 1 | 9.0 | 5.0 | .0 | 1 | 0 | 0 | .00 |
| 132 | 1 | 9.0 | 5.0 | .0 | 1 | 0 | 0 | .00 |
| 133 | 1 | 8.0 | 6.0 | 3.0 | 2 | 1 | 0 | .00 |
| 133 | 1 | 8.0 | 6.0 | 3.0 | 2 | 1 | 0 | .00 |
| 133 | 1 | 8.0 | 6.0 | 3.0 | 2 | 1 | 0 | .00 |
| 134 | 1 | 8.0 | 8.0 | 8.5 | 1 | 1 | 0 | .00 |
| 134 | 1 | 8.0 | 8.0 | 8.5 | 1 | 1 | 0 | .00 |
| 134 | 1 | 8.0 | 8.0 | 8.5 | 1 | 1 | 0 | .00 |
| 135 | 3 | 9.0 | 6.0 | 3.0 | 2 | 1 | 0 | 1.00 |
| 135 | 3 | 9.0 | 6.0 | 3.0 | 2 | 1 | 0 | .00 |
| 135 | 3 | 9.0 | 6.0 | 3.0 | 2 | 1 | 0 | .00 |
| 136 | 1 | 4.0 | 1.0 | 8.0 | 2 | 0 | 0 | 1.00 |
| 136 | 1 | 4.0 | 1.0 | 8.0 | 2 | 0 | 0 | .00 |
| 136 | 1 | 4.0 | 1.0 | 8.0 | 2 | 0 | 0 | .00 |
| 137 | 2 | 9.0 | .0 | 7.5 | 2 | 0 | 0 | .00 |
| 137 | 2 | 9.0 | .0 | 7.5 | 2 | 0 | 0 | .00 |
| 137 | 2 | 9.0 | .0 | 7.5 | 2 | 0 | 0 |  |
| 138 | 1 | 10.0 | 7.0 | 8.0 | 1 | 1 | 1 | .00 |
| 138 | 1 | 10.0 | 7.0 | 8.0 | 1 | 1 | 1 | .00 |
| 138 | 1 | 10.0 | 7.0 | 8.0 | 1 | 1 | 1 | .00 |
| 139 | 1 | 8.0 | 6.5 | 5.5 | 1 | 0 | 0 | .00 |
| 139 | 1 | 8.0 | 6.5 | 5.5 | 1 | 0 | 0 |  |
| 139 | 1 | 8.0 | 6.5 | 5.5 | 1 | 0 | 0 |  |
| 140 | 3 | 7.0 | 6.0 | 6.0 | 1 | 0 | 0 | .00 |
| 140 | 3 | 7.0 | 6.0 | 6.0 | 1 | 0 | 0 | .00 |
| 140 | 3 | 7.0 | 6.0 | 6.0 | 1 | 0 | 0 | .00 |
| 141 | 2 | 7.0 | 5.0 | 4.0 | 3 | 0 | 0 | .00 |
| 141 | 2 | 7.0 | 5.0 | 4.0 | 3 | 0 | 0 | .00 |
| 141 | 2 | 7.0 | 5.0 | 4.0 | 3 | 0 | 0 |  |
| 142 | 1 | 6.0 | 8.0 | 6.0 | 1 | 0 | 0 | .00 |
| 142 | 1 | 6.0 | 8.0 | 6.0 | 1 | 0 | 0 | .00 |
| 142 | 1 | 6.0 | 8.0 | 6.0 | 1 | 0 | 0 |  |
| 143 | 2 | 6.0 | 10.0 | 10.0 | 1 | 0 | 0 | .00 |
| 143 | 2 | 6.0 | 10.0 | 10.0 | 1 | 0 | 0 | .00 |
| 143 | 2 | 6.0 | 10.0 | 10.0 | 1 | 0 | 0 |  |
| 144 | 1 | 8.5 | 8.0 | 5.0 | 1 | 0 | 0 | .00 |
| 144 | 1 | 8.5 | 8.0 | 5.0 | 1 | 0 | 0 | .00 |
| 144 | 1 | 8.5 | 8.0 | 5.0 | 1 | 0 | 0 |  |
| 145 | 2 | 6.0 | 5.0 | 7.0 | 1 | 1 | 0 | .00 |
| 145 | 2 | 6.0 | 5.0 | 7.0 | 1 | 1 | 0 | .00 |
| 145 | 2 | 6.0 | 5.0 | 7.0 | 1 | 1 | 0 |  |
| 146 | 2 | 8.0 | 5.0 | 4.0 | 2 | 0 | 0 | .00 |
| 146 | 2 | 8.0 | 5.0 | 4.0 | 2 | 0 | 0 | .00 |
| 146 | 2 | 8.0 | 5.0 | 4.0 | 2 | 0 | 0 |  |
| 147 | 1 | 8.0 | 6.0 | 1.0 | 1 | 0 | 0 | .00 |
| 147 | 1 | 8.0 | 6.0 | 1.0 | 1 | 0 | 0 | .00 |
| 147 | 1 | 8.0 | 6.0 | 1.0 | 1 | 0 | 0 |  |
| 148 | 2 | 10.0 | 9.0 | 8.0 | 3 | 1 | 0 | .00 |
| 148 | 2 | 10.0 | 9.0 | 8.0 | 3 | 1 | 0 |  |
| 148 | 2 | 10.0 | 9.0 | 8.0 | 3 | 1 | 0 |  |
| 149 | 2 | 8.0 | 3.0 | 1.0 | 1 | 1 | 0 | .00 |
| 149 | 2 | 8.0 | 3.0 | 1.0 | 1 | 1 | 0 | .00 |
| 149 | 2 | 8.0 | 3.0 | 1.0 | 1 | 1 | 0 |  |
| 150 | 1 | 10.0 | 3.0 | 7.0 | 1 | 0 | 0 | .00 |
| 150 | 1 | 10.0 | 3.0 | 7.0 | 1 | 0 | 0 | .00 |
| 150 | 1 | 10.0 | 3.0 | 7.0 | 1 | 0 | 0 |  |
| 151 | 3 | 8.0 | .0 | 5.0 | 3 | 0 | 0 | .00 |
| 151 | 3 | 8.0 | .0 | 5.0 | 3 | 0 | 0 | .00 |
| 151 | 3 | 8.0 | .0 | 5.0 | 3 | 0 | 0 |  |
| 152 | 1 | 5.0 | 5.0 | 5.0 | 2 | 0 | 0 | .00 |
| 152 | 1 | 5.0 | 5.0 | 5.0 | 2 | 0 | 0 | .00 |
| 152 | 1 | 5.0 | 5.0 | 5.0 | 2 | 0 | 0 |  |
| 153 | 3 | 8.0 | 7.0 | .0 | 3 | 0 | 0 | .00 |
| 153 | 3 | 8.0 | 7.0 | .0 | 3 | 0 | 0 |  |
| 153 | 3 | 8.0 | 7.0 | .0 | 3 | 0 | 0 |  |
| 154 | 3 | 8.0 | 4.0 | 3.0 | 2 | 0 | 0 | .00 |
| 154 | 3 | 8.0 | 4.0 | 3.0 | 2 | 0 | 0 |  |
| 154 | 3 | 8.0 | 4.0 | 3.0 | 2 | 0 | 0 |  |
| 155 | 1 | 8.0 | 10.0 | 3.0 | 1 | 1 | 0 | .00 |
| 155 | 1 | 8.0 | 10.0 | 3.0 | 1 | 1 | 0 | 1.00 |
| 155 | 1 | 8.0 | 10.0 | 3.0 | 1 | 1 | 0 |  |
| 156 | 2 | 6.0 | 3.0 | 6.0 | 2 | 0 | 1 | .00 |
| 156 | 2 | 6.0 | 3.0 | 6.0 | 2 | 0 | 1 | .00 |
| 156 | 2 | 6.0 | 3.0 | 6.0 | 2 | 0 | 1 |  |
| 157 | 2 | 10.0 | 5.0 | .0 | 2 | 0 | 0 | .00 |
| 157 | 2 | 10.0 | 5.0 | .0 | 2 | 0 | 0 |  |
| 157 | 2 | 10.0 | 5.0 | .0 | 2 | 0 | 0 |  |
| 158 | 1 | 7.0 | 6.0 | 8.0 | 1 | 1 | 0 | .00 |
| 158 | 1 | 7.0 | 6.0 | 8.0 | 1 | 1 | 0 | .00 |
| 158 | 1 | 7.0 | 6.0 | 8.0 | 1 | 1 | 0 |  |
| 159 | 3 | 6.0 | 6.0 | 9.0 | 1 | 1 | 0 | .00 |
| 159 | 3 | 6.0 | 6.0 | 9.0 | 1 | 1 | 0 | .00 |
| 159 | 3 | 6.0 | 6.0 | 9.0 | 1 | 1 | 0 |  |
| 160 | 1 | 8.0 | 5.0 | 5.0 | 2 | 0 | 0 | .00 |
| 160 | 1 | 8.0 | 5.0 | 5.0 | 2 | 0 | 0 | .00 |
| 160 | 1 | 8.0 | 5.0 | 5.0 | 2 | 0 | 0 |  |
| 161 | 1 | 7.0 | 6.0 | 5.0 | 1 | 0 | 0 | .00 |
| 161 | 1 | 7.0 | 6.0 | 5.0 | 1 | 0 | 0 | .00 |
| 161 | 1 | 7.0 | 6.0 | 5.0 | 1 | 0 | 0 |  |
| 162 | 1 | 8.0 | 6.0 | 6.0 | 1 | 0 | 0 | .00 |
| 162 | 1 | 8.0 | 6.0 | 6.0 | 1 | 0 | 0 |  |
| 162 | 1 | 8.0 | 6.0 | 6.0 | 1 | 0 | 0 |  |
| 163 | 2 | 10.0 | 5.0 | 8.0 | 1 | 0 | 0 | .00 |
| 163 | 2 | 10.0 | 5.0 | 8.0 | 1 | 0 | 0 |  |
| 163 | 2 | 10.0 | 5.0 | 8.0 | 1 | 0 | 0 |  |
| 165 | 3 | 5.0 | .0 | 6.0 | 1 | 0 | 0 | .00 |
| 165 | 3 | 5.0 | .0 | 6.0 | 1 | 0 | 0 | .00 |
| 165 | 3 | 5.0 | .0 | 6.0 | 1 | 0 | 0 |  |
| 166 | 2 | 5.0 | 1.0 | 6.0 | 2 | 1 | 1 | .00 |
| 166 | 2 | 5.0 | 1.0 | 6.0 | 2 | 1 | 1 | .00 |
| 166 | 2 | 5.0 | 1.0 | 6.0 | 2 | 1 | 1 |  |
| 167 | 3 | 5.0 | 10.0 | 5.0 | 3 | 1 | 0 | .00 |
| 167 | 3 | 5.0 | 10.0 | 5.0 | 3 | 1 | 0 |  |
| 167 | 3 | 5.0 | 10.0 | 5.0 | 3 | 1 | 0 |  |
| 168 | 1 | 10.0 | 4.0 | 6.0 | 3 | 0 | 0 | .00 |
| 168 | 1 | 10.0 | 4.0 | 6.0 | 3 | 0 | 0 | .00 |
| 168 | 1 | 10.0 | 4.0 | 6.0 | 3 | 0 | 0 |  |
| 169 | 3 | 8.0 | 5.0 | 7.0 | 1 | 0 | 0 | .00 |
| 169 | 3 | 8.0 | 5.0 | 7.0 | 1 | 0 | 0 | .00 |
| 169 | 3 | 8.0 | 5.0 | 7.0 | 1 | 0 | 0 |  |
| 170 | 2 | 5.0 | .0 | 7.0 | 1 | 0 | 0 | .00 |
| 170 | 2 | 5.0 | .0 | 7.0 | 1 | 0 | 0 | 1.00 |
| 170 | 2 | 5.0 | .0 | 7.0 | 1 | 0 | 0 |  |
| 171 | 3 | 6.0 | 5.0 | 5.0 | 1 | 0 | 1 | .00 |
| 171 | 3 | 6.0 | 5.0 | 5.0 | 1 | 0 | 1 | .00 |
| 171 | 3 | 6.0 | 5.0 | 5.0 | 1 | 0 | 1 |  |
| 172 | 2 | 10.0 | 8.0 | .0 | 2 | 1 | 1 | .00 |
| 172 | 2 | 10.0 | 8.0 | .0 | 2 | 1 | 1 | .00 |
| 172 | 2 | 10.0 | 8.0 | .0 | 2 | 1 | 1 |  |
| 173 | 1 | 9.0 | 3.0 | 5.0 | 1 | 0 | 0 | .00 |
| 173 | 1 | 9.0 | 3.0 | 5.0 | 1 | 0 | 0 | .00 |
| 173 | 1 | 9.0 | 3.0 | 5.0 | 1 | 0 | 0 |  |
| 174 | 3 | 9.0 | .0 | 5.0 | 3 | 1 | 0 | .00 |
| 174 | 3 | 9.0 | .0 | 5.0 | 3 | 1 | 0 | .00 |
| 174 | 3 | 9.0 | .0 | 5.0 | 3 | 1 | 0 |  |
| 175 | 3 | 7.0 | 5.0 | 5.0 | 1 | 0 | 0 | .00 |
| 175 | 3 | 7.0 | 5.0 | 5.0 | 1 | 0 | 0 | .00 |
| 175 | 3 | 7.0 | 5.0 | 5.0 | 1 | 0 | 0 |  |
| 176 | 1 | 5.0 | 5.0 | .0 | 1 | 0 | 1 | .00 |
| 176 | 1 | 5.0 | 5.0 | .0 | 1 | 0 | 1 | .00 |
| 176 | 1 | 5.0 | 5.0 | .0 | 1 | 0 | 1 |  |
| 177 | 1 | 7.0 | 9.0 | 4.0 | 1 | 1 | 0 | .00 |
| 177 | 1 | 7.0 | 9.0 | 4.0 | 1 | 1 | 0 |  |
| 177 | 1 | 7.0 | 9.0 | 4.0 | 1 | 1 | 0 |  |
| 178 | 1 | 8.0 | .0 | 5.0 | 1 | 0 | 0 | .00 |
| 178 | 1 | 8.0 | .0 | 5.0 | 1 | 0 | 0 |  |
| 178 | 1 | 8.0 | .0 | 5.0 | 1 | 0 | 0 |  |
| 179 | 3 | 8.0 | 1.0 | 7.0 | 1 | 1 | 0 | .00 |
| 179 | 3 | 8.0 | 1.0 | 7.0 | 1 | 1 | 0 | .00 |
| 179 | 3 | 8.0 | 1.0 | 7.0 | 1 | 1 | 0 |  |
| 180 | 3 | 5.0 | 5.0 | 5.0 | 1 | 1 | 0 | .00 |
| 180 | 3 | 5.0 | 5.0 | 5.0 | 1 | 1 | 0 | .00 |
| 180 | 3 | 5.0 | 5.0 | 5.0 | 1 | 1 | 0 |  |
| 181 | 1 | 7.0 | 5.0 | 7.0 | 1 | 1 | 0 | .00 |
| 181 | 1 | 7.0 | 5.0 | 7.0 | 1 | 1 | 0 | .00 |
| 181 | 1 | 7.0 | 5.0 | 7.0 | 1 | 1 | 0 |  |
| 183 | 3 | 10.0 | 2.0 | 9.0 | 1 | 1 | 0 | .00 |
| 183 | 3 | 10.0 | 2.0 | 9.0 | 1 | 1 | 0 | .00 |
| 183 | 3 | 10.0 | 2.0 | 9.0 | 1 | 1 | 0 |  |
| 184 | 3 | 6.0 | 5.0 | 7.0 | 2 | 1 | 0 | .00 |
| 184 | 3 | 6.0 | 5.0 | 7.0 | 2 | 1 | 0 |  |
| 184 | 3 | 6.0 | 5.0 | 7.0 | 2 | 1 | 0 |  |
| 185 | 1 | 7.0 | 8.0 | 4.0 | 1 | 0 | 0 | .00 |
| 185 | 1 | 7.0 | 8.0 | 4.0 | 1 | 0 | 0 | .00 |
| 185 | 1 | 7.0 | 8.0 | 4.0 | 1 | 0 | 0 |  |
| 186 | 3 | 7.0 | 3.0 | 5.0 | 1 | 1 | 0 | .00 |
| 186 | 3 | 7.0 | 3.0 | 5.0 | 1 | 1 | 0 | .00 |
| 186 | 3 | 7.0 | 3.0 | 5.0 | 1 | 1 | 0 |  |
| 187 | 3 | 8.0 | 2.5 | 5.0 | 2 | 0 | 0 | .00 |
| 187 | 3 | 8.0 | 2.5 | 5.0 | 2 | 0 | 0 | .00 |
| 187 | 3 | 8.0 | 2.5 | 5.0 | 2 | 0 | 0 |  |
| 188 | 3 | 7.0 | 2.0 | 5.0 | 1 | 0 | 0 | .00 |
| 188 | 3 | 7.0 | 2.0 | 5.0 | 1 | 0 | 0 | .00 |
| 188 | 3 | 7.0 | 2.0 | 5.0 | 1 | 0 | 0 |  |
| 189 | 3 | 10.0 | 5.5 | 4.0 | 2 | 1 | 0 | .00 |
| 189 | 3 | 10.0 | 5.5 | 4.0 | 2 | 1 | 0 | .00 |
| 189 | 3 | 10.0 | 5.5 | 4.0 | 2 | 1 | 0 |  |
| 190 | 2 | 8.0 | 8.0 | 3.0 | 1 | 0 | 0 | .00 |
| 190 | 2 | 8.0 | 8.0 | 3.0 | 1 | 0 | 0 |  |
| 190 | 2 | 8.0 | 8.0 | 3.0 | 1 | 0 | 0 |  |
