# Supplementary material for: Sleep disturbance after acute coronary syndrome: A longitudinal study over 12 months
Source: PLoS One. 2022 Jun 3;17(6):e0269545. doi: 10.1371/journal.pone.0269545 (PMC9165780; doi:10.1371/journal.pone.0269545)
Supplement: S3 Dataset — (DOCX) [file pone.0269545.s003.docx]

| Participant number | Antidepressants0=no  1=yes | Body mass index | Current smoker  0=no  1=yes | Alcohol consumption  0=moderate  1=none  2=heavy | Sports that make you sweat  0=none  1=1-2x/week  2=3-7x/week |  |
| --- | --- | --- | --- | --- | --- | --- |
| 2 | 0 | 25.00 | 1 | 1.00 | 0 |  |
| 2 | 0 | 23.30 | 0 | 1.00 | 2 |  |
| 2 | 0 | 22.60 | 0 | 1.00 | 1 |  |
| 3 | 0 | 15.90 | 0 | .00 | 1 |  |
| 3 | 0 | 15.40 | 0 | .00 | 2 |  |
| 3 | 0 | 15.20 | 0 | .00 | 1 |  |
| 4 | 0 | 22.50 | 1 | .00 | 0 |  |
| 4 | 0 | 23.50 | 0 | .00 | 2 |  |
| 4 |  |  |  |  |  |  |
| 5 | 0 | 24.80 | 1 | .00 | 2 |  |
| 5 | 0 | 23.60 | 0 | .00 | 1 |  |
| 5 | 0 | 24.20 | 0 | .00 | 1 |  |
| 6 | 0 | 34.56 | 1 | 1.00 | 0 |  |
| 6 | 1 | 33.10 | 1 | 1.00 | 0 |  |
| 6 | 1 | 31.60 | 1 | 1.00 | 2 |  |
| 7 | 0 | 25.35 | 0 | .00 | 2 |  |
| 7 | 0 | 32.90 | 0 | .00 | 2 |  |
| 7 | 0 | 32.10 | 0 | .00 | 2 |  |
| 8 | 0 | 31.90 | 0 | .00 | 2 |  |
| 8 | 0 | 31.40 | 0 | .00 | 2 |  |
| 8 | 0 | 34.30 | 0 | .00 | 2 |  |
| 9 | 0 | 36.40 | 0 | .00 | 1 |  |
| 9 | 0 | 35.30 | 0 | .00 | 1 |  |
| 9 | 0 | 35.40 | 0 | .00 | 1 |  |
| 10 | 0 | 28.70 | 0 | .00 | 1 |  |
| 10 | 0 | 24.80 | 0 | .00 | 0 |  |
| 10 | 0 | 24.90 | 0 | .00 | 2 |  |
| 11 | 1 | 25.20 | 0 | 1.00 | 2 |  |
| 11 | 1 | 24.80 | 0 | 1.00 | 1 |  |
| 11 | 1 | 24.50 | 0 | 1.00 | 2 |  |
| 12 | 0 | 30.00 | 0 | .00 | 2 |  |
| 12 |  |  |  |  |  |  |
| 12 |  |  |  |  |  |  |
| 13 | 0 | 30.10 | 0 | .00 | 0 |  |
| 13 |  |  |  |  |  |  |
| 13 |  |  |  |  |  |  |
| 14 | 0 | 27.80 | 0 | .00 | 0 |  |
| 14 | 0 | 25.00 | 0 | .00 | 1 |  |
| 14 | 0 | 25.90 | 0 | .00 | 0 |  |
| 16 | 0 | 27.90 | 1 | 1.00 | 0 |  |
| 16 |  |  |  |  |  |  |
| 16 |  |  |  |  |  |  |
| 17 | 0 | 26.80 | 0 | .00 | 1 |  |
| 17 | 1 | 26.80 | 0 | .00 | 0 |  |
| 17 | 1 | 29.00 | 0 | .00 | 0 |  |
| 18 | 0 | 28.00 | 1 | 2.00 | 0 |  |
| 18 |  |  |  |  |  |  |
| 18 |  |  |  |  |  |  |
| 19 | 0 | 21.60 | 1 | 1.00 | 0 |  |
| 19 | 0 | 21.10 | 1 | 1.00 | 0 |  |
| 19 | 0 | 22.20 | 1 | 1.00 | 2 |  |
| 21 | 0 | 23.60 | 1 | 1.00 | 0 |  |
| 21 | 0 | 22.30 | 0 | 1.00 | 0 |  |
| 21 | 0 |  | 0 | 1.00 | 2 |  |
| 22 | 0 | 33.10 | 0 | .00 | 1 |  |
| 22 | 0 | 32.80 | 0 | .00 | 2 |  |
| 22 | 0 | 33.16 | 0 | .00 | 2 |  |
| 24 | 0 | 35.60 | 1 | 1.00 | 0 |  |
| 24 | 0 |  | 0 | 1.00 | 0 |  |
| 24 | 0 |  | 0 | 1.00 | 0 |  |
| 25 | 0 | 34.10 | 0 | .00 | 2 |  |
| 25 | 0 | 29.40 | 0 | .00 | 2 |  |
| 25 | 0 | 28.00 | 0 | .00 | 2 |  |
| 26 | 0 | 28.10 | 0 | .00 | 0 |  |
| 26 | 0 | 29.40 | 0 | .00 | 0 |  |
| 26 | 0 | 27.50 | 0 | .00 | 1 |  |
| 27 | 1 | 33.70 | 0 | .00 | 0 |  |
| 27 |  |  |  |  |  |  |
| 27 |  |  |  |  |  |  |
| 28 | 0 | 24.50 | 1 | .00 | 0 |  |
| 28 | 0 | 25.20 | 0 | .00 | 1 |  |
| 28 | 0 | 24.97 | 0 | .00 | 2 |  |
| 29 | 0 | 29.60 | 0 | .00 | 1 |  |
| 29 | 0 | 30.20 | 0 | .00 | 1 |  |
| 29 | 0 | 31.10 | 0 | .00 | 1 |  |
| 30 | 0 | 31.00 | 0 | .00 | 0 |  |
| 30 | 0 | 30.00 | 0 | .00 | 1 |  |
| 30 | 0 | 32.20 | 0 | .00 | 0 |  |
| 31 | 0 | 29.40 | 0 | .00 | 0 |  |
| 31 | 0 | 29.90 | 0 | .00 | 0 |  |
| 31 | 0 | 26.00 | 0 | .00 | 0 |  |
| 32 | 0 | 24.40 | 1 | .00 | 1 |  |
| 32 | 0 | 24.20 | 0 | .00 | 2 |  |
| 32 | 0 | 24.40 | 0 | .00 | 2 |  |
| 33 | 1 | 21.90 | 0 | .00 | 1 |  |
| 33 |  |  |  |  |  |  |
| 33 |  |  |  |  |  |  |
| 34 | 0 | 23.50 | 1 | .00 | 0 |  |
| 34 | 1 | 24.50 | 1 | .00 | 1 |  |
| 34 | 1 | 25.60 | 1 | .00 | 1 |  |
| 35 | 0 | 24.80 | 1 | .00 | 1 |  |
| 35 | 0 | 21.90 | 0 | .00 | 2 |  |
| 35 |  |  |  |  |  |  |
| 36 | 0 | 27.30 | 1 | .00 | 0 |  |
| 36 |  |  |  |  |  |  |
| 36 |  |  |  |  |  |  |
| 37 | 0 | 37.90 | 0 | 1.00 | 0 |  |
| 37 |  |  |  |  |  |  |
| 37 |  |  |  |  |  |  |
| 38 | 0 | 23.90 | 0 | .00 | 1 |  |
| 38 | 0 | 22.20 | 0 | .00 | 2 |  |
| 38 | 0 | 22.77 | 0 | .00 | 2 |  |
| 39 | 0 | 22.00 | 1 | .00 | 1 |  |
| 39 | 0 |  | 0 | .00 | 2 |  |
| 39 | 0 |  | 0 | .00 | 1 |  |
| 40 | 0 | 29.70 | 1 | .00 | 0 |  |
| 40 | 0 |  | 1 | .00 | 2 |  |
| 40 | 0 | 29.70 | 1 | 2.00 | 0 |  |
| 41 | 0 | 33.60 | 1 | 1.00 | 0 |  |
| 41 | 0 | 34.70 | 0 | 1.00 | 0 |  |
| 41 | 0 | 33.80 | 0 | 1.00 | 0 |  |
| 42 | 0 | 27.20 | 0 | .00 | 1 |  |
| 42 | 0 | 25.40 | 0 | .00 | 2 |  |
| 42 | 0 | 24.50 | 0 | .00 | 2 |  |
| 43 | 0 | 25.10 | 0 | .00 | 2 |  |
| 43 | 0 | 26.50 | 0 | .00 | 2 |  |
| 43 | 0 | 25.68 | 0 | .00 | 2 |  |
| 44 | 0 | 35.70 | 0 | .00 | 1 |  |
| 44 | 0 | 36.40 | 0 | 2.00 | 2 |  |
| 44 | 0 | 37.10 | 0 | .00 | 2 |  |
| 45 | 1 | 40.60 | 0 | .00 | 0 |  |
| 45 | 1 | 34.80 | 0 | .00 | 2 |  |
| 45 | 1 | 33.13 | 0 | .00 | 2 |  |
| 46 | 0 | 29.20 | 0 | .00 | 2 |  |
| 46 | 0 | 28.50 | 0 | .00 | 2 |  |
| 46 | 0 | 28.90 | 0 | .00 | 2 |  |
| 47 | 1 | 25.00 | 1 | 2.00 | 2 |  |
| 47 | 0 | 23.50 | 1 | 2.00 | 2 |  |
| 47 |  |  |  |  |  |  |
| 48 | 0 | 38.10 | 1 | .00 | 1 |  |
| 48 | 0 | 39.20 | 0 | .00 | 2 |  |
| 48 | 0 | 39.20 | 1 | .00 | 1 |  |
| 49 | 0 | 24.10 | 0 | .00 | 1 |  |
| 49 | 0 | 22.80 | 0 | .00 | 2 |  |
| 49 | 1 | 23.20 | 0 | .00 | 2 |  |
| 50 | 0 | 22.60 | 0 | 2.00 | 0 |  |
| 50 | 0 | 22.80 | 0 | 2.00 | 2 |  |
| 50 | 0 | 22.50 | 0 | 2.00 | 2 |  |
| 51 | 0 | 27.50 | 0 | .00 | 2 |  |
| 51 | 1 | 26.50 | 0 | .00 | 0 |  |
| 51 | 1 | 26.40 | 0 | .00 | 1 |  |
| 52 | 0 | 29.10 | 1 | .00 | 2 |  |
| 52 |  |  |  |  |  |  |
| 52 |  |  |  |  |  |  |
| 53 | 0 | 24.70 | 0 | .00 | 2 |  |
| 53 | 0 | 25.20 | 0 | .00 | 2 |  |
| 53 | 0 | 24.00 | 0 | .00 | 2 |  |
| 54 | 0 | 39.60 | 0 | .00 | 2 |  |
| 54 | 0 | 36.70 | 0 | .00 | 2 |  |
| 54 | 0 |  | 0 | .00 | 1 |  |
| 55 | 0 | 23.50 | 0 | .00 | 2 |  |
| 55 | 0 | 23.00 | 0 | 2.00 | 2 |  |
| 55 | 0 | 22.80 | 0 | .00 | 2 |  |
| 56 | 0 | 22.60 | 0 | 1.00 | 2 |  |
| 56 | 0 | 24.30 | 0 | 1.00 | 2 |  |
| 56 | 0 | 24.60 | 0 | 1.00 | 2 |  |
| 57 | 0 | 19.40 | 0 | .00 | 1 |  |
| 57 | 0 | 18.90 | 0 | .00 | 2 |  |
| 57 | 0 | 19.20 | 0 | .00 | 2 |  |
| 58 | 0 | 25.20 | 0 | .00 | 1 |  |
| 58 | 0 | 24.60 | 0 | .00 | 1 |  |
| 58 | 0 | 24.60 | 0 | .00 | 2 |  |
| 59 | 0 | 37.70 | 1 | .00 | 0 |  |
| 59 | 0 | 38.00 | 1 | .00 | 2 |  |
| 59 | 0 | 38.20 | 0 | .00 | 2 |  |
| 60 | 0 | 28.40 | 0 | .00 | 0 |  |
| 60 | 0 | 27.70 | 0 | 1.00 | 2 |  |
| 60 | 0 | 29.70 | 0 | 1.00 | 0 |  |
| 61 | 0 | 29.40 | 0 | .00 | 0 |  |
| 61 |  |  |  |  |  |  |
| 61 |  |  |  |  |  |  |
| 62 | 0 | 29.50 | 1 | .00 | 0 |  |
| 62 |  |  |  |  |  |  |
| 62 |  |  |  |  |  |  |
| 63 | 0 | 39.10 | 1 | .00 | 0 |  |
| 63 | 0 | 41.20 | 0 | .00 | 2 |  |
| 63 |  |  |  |  |  |  |
| 64 | 0 | 30.00 | 0 | .00 | 0 |  |
| 64 | 0 | 28.10 | 0 | .00 | 2 |  |
| 64 | 1 | 27.10 | 0 | .00 | 2 |  |
| 65 | 0 | 25.10 | 0 | .00 | 1 |  |
| 65 | 0 | 30.40 | 0 | .00 | 1 |  |
| 65 | 0 | 30.00 | 0 | .00 | 1 |  |
| 66 | 0 | 30.10 | 1 | 1.00 | 0 |  |
| 66 | 0 | 31.80 | 0 | 1.00 | 2 |  |
| 66 | 0 |  | 0 | 1.00 | 0 |  |
| 67 | 0 | 32.30 | 1 | .00 | 0 |  |
| 67 | 0 | 30.00 | 0 | .00 | 2 |  |
| 67 | 1 | 28.70 | 0 | .00 | 2 |  |
| 68 | 0 | 24.80 | 1 | .00 | 1 |  |
| 68 | 0 | 27.80 | 0 | .00 | 2 |  |
| 68 | 0 | 27.70 | 0 | .00 | 1 |  |
| 69 | 0 | 27.70 | 0 | .00 | 0 |  |
| 69 | 0 | 25.60 | 0 | .00 | 0 |  |
| 69 |  |  |  |  |  |  |
| 72 | 0 | 23.30 | 0 | .00 | 1 |  |
| 72 | 0 | 24.90 | 0 | .00 | 2 |  |
| 72 | 0 | 22.90 | 0 | .00 | 2 |  |
| 73 | 0 | 27.40 | 0 | .00 | 2 |  |
| 73 | 0 | 28.00 | 0 | .00 | 2 |  |
| 73 | 0 | 28.40 | 0 | .00 | 2 |  |
| 74 | 0 | 23.80 | 1 | 1.00 | 0 |  |
| 74 |  |  |  |  |  |  |
| 74 |  |  |  |  |  |  |
| 75 | 1 | 29.90 | 0 | .00 | 2 |  |
| 75 |  |  |  |  |  |  |
| 75 |  |  |  |  |  |  |
| 76 | 0 | 25.90 | 0 | 1.00 | 0 |  |
| 76 |  |  |  |  |  |  |
| 76 | 0 |  | 0 | 1.00 | 0 |  |
| 77 | 0 | 29.00 | 1 | .00 | 1 |  |
| 77 |  |  |  |  |  |  |
| 77 |  |  |  |  |  |  |
| 78 | 1 | 25.40 | 1 | .00 | 2 |  |
| 78 | 1 | 26.30 | 0 | .00 | 2 |  |
| 78 | 1 | 29.10 | 0 | .00 | 0 |  |
| 80 | 1 | 34.00 | 1 | .00 | 2 |  |
| 80 |  |  |  |  |  |  |
| 80 |  |  |  |  |  |  |
| 81 | 0 | 25.30 | 0 | .00 | 2 |  |
| 81 | 0 | 25.60 | 0 | .00 | 2 |  |
| 81 | 0 | 25.70 | 0 | .00 | 2 |  |
| 82 | 0 | 25.00 | 0 | 2.00 | 2 |  |
| 82 |  |  |  |  |  |  |
| 82 | 0 | 24.10 | 0 | .00 | 1 |  |
| 83 | 0 | 34.70 | 1 | .00 | 0 |  |
| 83 | 0 | 34.70 | 0 | .00 | 0 |  |
| 83 | 0 | 32.60 | 0 | .00 | 0 |  |
| 84 | 0 | 29.30 | 0 | 1.00 | 0 |  |
| 84 | 0 | 28.80 | 0 | .00 | 0 |  |
| 84 | 0 | 28.40 | 0 | 1.00 | 0 |  |
| 85 | 0 | 27.10 | 1 | 1.00 | 1 |  |
| 85 | 0 | 26.40 | 1 | 1.00 | 0 |  |
| 85 | 0 | 27.20 | 1 | 1.00 | 0 |  |
| 86 | 0 | 28.10 | 1 | .00 | 0 |  |
| 86 | 0 | 28.10 | 0 | .00 | 0 |  |
| 86 | 0 | 28.10 | 0 | 1.00 | 0 |  |
| 87 | 0 | 24.30 | 0 | .00 | 0 |  |
| 87 | 0 | 23.40 | 0 | 2.00 | 0 |  |
| 87 | 0 | 24.10 | 0 | .00 | 0 |  |
| 88 | 0 | 26.90 | 0 | .00 | 0 |  |
| 88 | 0 | 24.60 | 0 | .00 | 2 |  |
| 88 | 0 | 24.40 | 0 | .00 | 2 |  |
| 90 | 0 | 37.00 | 1 | .00 | 1 |  |
| 90 | 0 | 37.90 | 0 | .00 | 2 |  |
| 90 | 0 | 39.80 | 0 | 1.00 | 1 |  |
| 91 | 0 | 22.10 | 1 | .00 | 1 |  |
| 91 | 0 | 22.60 | 0 | 1.00 | 1 |  |
| 91 | 0 | 23.60 | 0 | .00 | 1 |  |
| 92 | 0 | 25.50 | 0 | 1.00 | 0 |  |
| 92 | 0 |  | 0 | 1.00 | 0 |  |
| 92 | 0 | 21.20 | 0 | 1.00 | 0 |  |
| 93 | 0 | 24.80 | 1 | .00 | 1 |  |
| 93 | 0 | 25.40 | 0 | .00 | 2 |  |
| 93 | 0 | 25.40 | 0 | .00 | 2 |  |
| 94 | 0 | 23.00 | 0 | .00 | 1 |  |
| 94 | 0 | 23.40 | 0 | .00 | 2 |  |
| 94 | 0 | 23.20 | 0 | .00 | 2 |  |
| 95 | 0 | 24.70 | 0 | .00 | 2 |  |
| 95 | 0 |  | 0 | .00 | 2 |  |
| 95 |  |  |  |  |  |  |
| 96 | 1 | 21.60 | 1 | .00 | 0 |  |
| 96 |  |  |  |  |  |  |
| 96 |  |  |  |  |  |  |
| 97 | 0 | 30.10 | 1 | 2.00 | 0 |  |
| 97 | 0 | 29.20 | 0 | 2.00 | 2 |  |
| 97 | 0 | 28.30 | 0 | 2.00 | 0 |  |
| 98 | 0 | 27.60 | 0 | 2.00 | 2 |  |
| 98 | 0 | 26.85 | 0 | 2.00 | 2 |  |
| 98 | 0 | 27.70 | 0 | .00 | 2 |  |
| 99 | 0 | 30.90 | 0 | .00 | 0 |  |
| 99 | 0 | 29.60 | 0 | .00 | 0 |  |
| 99 | 0 | 30.10 | 0 | .00 | 1 |  |
| 100 | 0 | 32.80 | 1 | .00 | 2 |  |
| 100 | 0 | 32.40 | 0 | .00 | 2 |  |
| 100 | 0 | 34.30 | 1 | .00 | 2 |  |
| 101 | 0 | 23.10 | 1 | .00 | 0 |  |
| 101 | 1 | 21.40 | 1 | .00 | 0 |  |
| 101 | 1 | 22.10 | 0 | .00 | 0 |  |
| 102 | 0 | 30.80 | 1 | .00 | 0 |  |
| 102 | 0 | 28.60 | 1 | .00 | 0 |  |
| 102 | 0 | 31.10 | 1 | .00 | 2 |  |
| 103 | 0 | 18.60 | 0 | 1.00 | 0 |  |
| 103 |  |  |  |  |  |  |
| 103 |  |  |  |  |  |  |
| 104 | 0 | 24.10 | 0 | .00 | 0 |  |
| 104 | 0 | 23.30 | 0 | .00 | 0 |  |
| 104 | 0 | 22.50 | 0 | .00 | 0 |  |
| 105 | 0 | 24.70 | 1 | .00 | 0 |  |
| 105 |  |  |  |  |  |  |
| 105 |  |  |  |  |  |  |
| 106 | 0 | 34.30 | 1 | .00 | 0 |  |
| 106 | 0 | 36.20 | 0 | .00 | 2 |  |
| 106 | 1 | 34.90 | 0 | 1.00 | 1 |  |
| 107 | 0 | 29.10 | 0 | .00 | 2 |  |
| 107 | 0 | 29.40 | 0 | .00 | 2 |  |
| 107 | 1 | 30.40 | 0 | .00 | 2 |  |
| 108 | 0 | 31.40 | 1 | .00 | 0 |  |
| 108 | 0 | 30.10 | 0 | 1.00 | 2 |  |
| 108 | 0 | 30.05 | 0 | 1.00 | 1 |  |
| 109 | 0 | 24.50 | 1 | .00 | 0 |  |
| 109 | 0 | 25.20 | 0 | .00 | 2 |  |
| 109 | 0 | 25.14 | 0 | .00 | 2 |  |
| 110 | 0 | 30.50 | 0 | .00 | 0 |  |
| 110 |  |  |  |  |  |  |
| 110 |  |  |  |  |  |  |
| 111 | 0 | 42.97 | 0 | .00 | 0 |  |
| 111 | 0 | 43.50 | 0 | .00 | 2 |  |
| 111 | 0 | 45.80 | 0 | .00 | 1 |  |
| 112 | 0 | 26.60 | 0 | .00 | 1 |  |
| 112 | 0 | 24.10 | 0 | .00 | 2 |  |
| 112 |  |  |  |  |  |  |
| 113 | 0 | 27.10 | 0 | .00 | 2 |  |
| 113 | 0 | 25.90 | 0 | .00 | 2 |  |
| 113 | 0 | 26.47 | 0 | .00 | 2 |  |
| 114 | 0 | 24.10 | 0 | 1.00 | 1 |  |
| 114 | 0 | 23.20 | 0 | 1.00 | 2 |  |
| 114 | 0 | 22.80 | 0 | 1.00 | 2 |  |
| 115 | 0 | 28.40 | 1 | .00 | 2 |  |
| 115 | 0 | 28.80 | 0 | .00 | 2 |  |
| 115 | 0 | 28.90 | 0 | .00 | 2 |  |
| 116 | 0 | 26.90 | 1 | 1.00 | 0 |  |
| 116 | 1 | 30.90 | 0 | 1.00 | 2 |  |
| 116 | 0 | 28.00 | 0 | 1.00 | 2 |  |
| 117 | 0 | 33.10 | 0 | .00 | 0 |  |
| 117 | 0 | 33.30 | 0 | .00 | 0 |  |
| 117 | 0 | 33.70 | 0 | .00 | 0 |  |
| 118 | 0 | 21.90 | 0 | .00 | 0 |  |
| 118 |  |  |  |  |  |  |
| 118 |  |  |  |  |  |  |
| 119 | 0 | 21.60 | 0 | 2.00 | 2 |  |
| 119 | 1 | 21.60 | 0 | .00 | 2 |  |
| 119 | 1 | 22.20 | 0 | .00 | 2 |  |
| 120 | 0 | 25.60 | 1 | .00 | 0 |  |
| 120 | 0 | 26.35 | 0 | 1.00 | 0 |  |
| 120 | 0 | 24.20 | 1 | 1.00 | 0 |  |
| 121 | 0 | 31.20 | 1 | .00 | 0 |  |
| 121 | 0 | 34.10 | 1 | .00 | 0 |  |
| 121 | 0 | 31.60 | 1 | .00 | 2 |  |
| 122 | 0 | 21.60 | 1 | .00 | 0 |  |
| 122 |  |  |  |  |  |  |
| 122 |  |  |  |  |  |  |
| 123 | 0 | 27.20 | 0 | .00 | 2 |  |
| 123 | 0 | 23.95 | 0 | .00 | 2 |  |
| 123 | 0 | 24.00 | 0 | 2.00 | 2 |  |
| 124 | 0 | 22.10 | 0 | .00 | 0 |  |
| 124 |  |  |  |  |  |  |
| 124 |  |  |  |  |  |  |
| 125 | 0 | 25.70 | 0 | .00 | 0 |  |
| 125 | 0 | 25.80 | 0 | 2.00 | 2 |  |
| 125 | 0 | 25.20 | 0 | .00 | 1 |  |
| 126 | 0 | 23.00 | 0 | .00 | 2 |  |
| 126 | 0 | 23.00 | 0 | .00 | 2 |  |
| 126 | 0 | 23.50 | 0 | .00 | 2 |  |
| 127 | 0 | 26.90 | 0 | .00 | 1 |  |
| 127 | 0 | 26.00 | 0 | .00 | 1 |  |
| 127 | 0 | 25.30 | 0 | .00 | 1 |  |
| 128 | 0 | 25.95 | 1 | .00 | 2 |  |
| 128 | 0 | 27.00 | 0 | .00 | 2 |  |
| 128 | 0 | 28.40 | 0 | 2.00 | 1 |  |
| 129 | 0 | 35.40 | 1 | .00 | 0 |  |
| 129 | 0 | 35.00 | 1 | .00 | 0 |  |
| 129 | 0 | 35.70 | 1 | .00 | 0 |  |
| 130 | 0 | 19.60 | 1 | .00 | 0 |  |
| 130 | 0 | 21.10 | 0 | .00 | 2 |  |
| 130 | 1 | 21.70 | 1 | .00 | 0 |  |
| 131 | 0 | 27.90 | 0 | .00 | 0 |  |
| 131 | 0 | 27.10 | 0 | .00 | 2 |  |
| 131 | 0 | 27.40 | 0 | .00 | 1 |  |
| 132 | 0 | 25.80 | 0 | .00 | 1 |  |
| 132 | 0 | 26.10 | 0 | .00 | 2 |  |
| 132 | 0 | 29.10 | 0 | .00 | 1 |  |
| 133 | 1 | 30.20 | 0 | .00 | 1 |  |
| 133 | 1 | 30.70 | 0 | 1.00 | 2 |  |
| 133 | 1 | 32.00 | 0 | .00 | 2 |  |
| 134 | 0 | 27.80 | 1 | .00 | 0 |  |
| 134 | 0 | 27.40 | 0 | .00 | 2 |  |
| 134 | 0 | 27.10 | 1 | .00 | 0 |  |
| 135 | 0 | 25.20 | 1 | .00 | 0 |  |
| 135 | 0 | 23.30 | 0 | .00 | 2 |  |
| 135 | 0 | 24.60 | 0 | .00 | 0 |  |
| 136 | 0 | 27.20 | 0 | .00 | 1 |  |
| 136 | 0 | 25.30 | 0 | .00 | 2 |  |
| 136 | 0 | 25.90 | 0 | .00 | 2 |  |
| 137 | 0 | 32.20 | 1 | .00 | 2 |  |
| 137 | 0 | 35.30 | 0 | .00 | 2 |  |
| 137 |  |  |  |  |  |  |
| 138 | 0 | 33.70 | 0 | .00 | 0 |  |
| 138 | 0 | 34.00 | 0 | .00 | 2 |  |
| 138 | 0 | 35.60 | 0 | .00 | 0 |  |
| 139 | 0 | 30.40 | 0 | .00 | 2 |  |
| 139 |  |  |  |  |  |  |
| 139 |  |  |  |  |  |  |
| 140 | 0 | 25.60 | 0 | .00 | 2 |  |
| 140 | 0 | 23.30 | 0 | .00 | 2 |  |
| 140 | 0 | 24.10 | 0 | .00 | 1 |  |
| 141 | 0 | 27.70 | 0 | .00 | 1 |  |
| 141 | 0 | 28.70 | 0 | .00 | 1 |  |
| 141 |  |  |  |  |  |  |
| 142 | 0 | 22.20 | 0 | 1.00 | 1 |  |
| 142 | 0 | 22.20 | 0 | 1.00 | 1 |  |
| 142 |  |  |  |  |  |  |
| 143 | 0 | 25.50 | 1 | .00 | 0 |  |
| 143 | 0 | 22.50 | 0 | 1.00 | 2 |  |
| 143 |  |  |  |  |  |  |
| 144 | 0 | 28.70 | 1 | 2.00 | 1 |  |
| 144 | 0 | 29.40 | 0 | .00 | 2 |  |
| 144 |  |  |  |  |  |  |
| 145 | 0 | 25.10 | 1 | .00 | 2 |  |
| 145 | 0 | 26.20 | 1 | .00 | 2 |  |
| 145 |  |  |  |  |  |  |
| 146 | 0 | 25.90 | 1 | 1.00 | 1 |  |
| 146 | 0 | 22.04 | 0 | 1.00 | 1 |  |
| 146 |  |  |  |  |  |  |
| 147 | 0 | 19.70 | 1 | .00 | 1 |  |
| 147 | 0 | 25.10 | 0 | 1.00 | 1 |  |
| 147 |  |  |  |  |  |  |
| 148 | 0 | 35.10 | 1 | 1.00 | 0 |  |
| 148 |  |  |  |  |  |  |
| 148 |  |  |  |  |  |  |
| 149 | 1 | 27.20 | 0 | 1.00 | 0 |  |
| 149 | 1 | 20.20 | 0 | 1.00 | 2 |  |
| 149 |  |  |  |  |  |  |
| 150 | 0 | 27.40 | 1 | .00 | 0 |  |
| 150 | 0 | 27.10 | 0 | .00 | 2 |  |
| 150 |  |  |  |  |  |  |
| 151 | 0 | 27.00 | 0 | 1.00 | 1 |  |
| 151 | 0 | 26.60 | 0 | .00 | 1 |  |
| 151 |  |  |  |  |  |  |
| 152 | 0 | 34.70 | 1 | .00 | 0 |  |
| 152 | 0 | 33.20 | 0 | .00 | 2 |  |
| 152 |  |  |  |  |  |  |
| 153 | 0 | 25.50 | 0 | 1.00 | 1 |  |
| 153 |  |  |  |  |  |  |
| 153 |  |  |  |  |  |  |
| 154 | 1 | 23.40 | 1 | .00 | 0 |  |
| 154 |  | 21.30 | 1 | .00 | 2 |  |
| 154 |  |  |  |  |  |  |
| 155 | 0 | 36.00 | 1 | .00 | 2 |  |
| 155 | 0 | 36.30 | 1 | 1.00 | 0 |  |
| 155 |  |  |  |  |  |  |
| 156 | 0 | 27.40 | 0 | 1.00 | 1 |  |
| 156 | 0 | 24.10 | 0 | 1.00 | 2 |  |
| 156 |  |  |  |  |  |  |
| 157 | 0 | 23.50 | 0 | .00 | 0 |  |
| 157 |  |  |  |  |  |  |
| 157 |  |  |  |  |  |  |
| 158 | 0 | 21.40 | 1 | .00 | 2 |  |
| 158 | 0 | 20.70 | 0 | 1.00 | 0 |  |
| 158 |  |  |  |  |  |  |
| 159 | 0 | 26.20 | 1 | .00 | 1 |  |
| 159 | 0 | 25.20 | 1 | .00 | 1 |  |
| 159 |  |  |  |  |  |  |
| 160 | 0 | 32.50 | 1 | .00 | 2 |  |
| 160 | 0 | 28.00 | 0 | .00 | 2 |  |
| 160 |  |  |  |  |  |  |
| 161 | 0 | 25.40 | 0 | .00 | 1 |  |
| 161 | 0 | 25.20 | 0 | .00 | 2 |  |
| 161 |  |  |  |  |  |  |
| 162 | 0 | 28.40 | 0 | .00 | 1 |  |
| 162 |  |  |  |  |  |  |
| 162 |  |  |  |  |  |  |
| 163 | 0 | 26.90 | 1 | .00 | 0 |  |
| 163 |  |  |  |  |  |  |
| 163 |  |  |  |  |  |  |
| 165 | 0 | 25.00 | 0 | .00 | 2 |  |
| 165 | 0 | 24.00 | 0 | .00 | 2 |  |
| 165 |  |  |  |  |  |  |
| 166 | 1 | 34.60 | 1 | 1.00 | 0 |  |
| 166 | 1 | 37.10 | 1 | 1.00 | 0 |  |
| 166 |  |  |  |  |  |  |
| 167 | 0 | 19.21 | 1 | .00 | 1 |  |
| 167 |  |  |  |  |  |  |
| 167 |  |  |  |  |  |  |
| 168 | 0 | 27.10 | 0 | .00 | 2 |  |
| 168 | 0 | 27.80 | 0 | .00 | 2 |  |
| 168 |  |  |  |  |  |  |
| 169 | 0 | 33.30 | 1 | 2.00 | 0 |  |
| 169 | 0 | 32.00 | 0 | .00 | 2 |  |
| 169 |  |  |  |  |  |  |
| 170 | 0 | 25.20 | 1 | 1.00 | 2 |  |
| 170 | 0 | 24.82 | 1 | .00 | 2 |  |
| 170 |  |  |  |  |  |  |
| 171 | 1 | 25.40 | 0 | .00 | 2 |  |
| 171 | 0 | 24.05 | 0 | .00 | 2 |  |
| 171 |  |  |  |  |  |  |
| 172 | 1 | 29.00 | 0 | .00 | 0 |  |
| 172 | 0 | 29.02 | 0 | .00 | 2 |  |
| 172 |  |  |  |  |  |  |
| 173 | 0 | 22.40 | 0 | 1.00 | 1 |  |
| 173 | 0 | 22.10 | 0 | 1.00 | 0 |  |
| 173 |  |  |  |  |  |  |
| 174 | 0 | 30.90 | 0 | .00 | 2 |  |
| 174 | 0 | 30.60 | 0 | 1.00 | 2 |  |
| 174 |  |  |  |  |  |  |
| 175 | 0 | 27.50 | 0 | .00 | 1 |  |
| 175 | 0 | 25.30 | 0 | .00 | 2 |  |
| 175 |  |  |  |  |  |  |
| 176 | 0 | 32.20 | 0 | .00 | 1 |  |
| 176 | 0 | 31.20 | 0 | .00 | 2 |  |
| 176 |  |  |  |  |  |  |
| 177 | 1 | 24.90 | 0 | .00 | 0 |  |
| 177 |  |  |  |  |  |  |
| 177 |  |  |  |  |  |  |
| 178 | 0 | 26.20 | 0 | .00 | 0 |  |
| 178 |  | 25.90 | 0 | .00 | 1 |  |
| 178 |  |  |  |  |  |  |
| 179 | 0 | 28.70 | 1 | .00 | 0 |  |
| 179 | 0 | 28.80 | 0 | .00 | 2 |  |
| 179 |  |  |  |  |  |  |
| 180 | 0 | 22.90 | 0 | 1.00 | 2 |  |
| 180 | 0 | 23.10 | 0 | 1.00 | 2 |  |
| 180 |  |  |  |  |  |  |
| 181 | 0 | 25.90 | 1 | .00 | 0 |  |
| 181 | 1 | 27.00 | 0 | .00 | 2 |  |
| 181 |  |  |  |  |  |  |
| 183 | 0 | 21.30 | 1 | 1.00 | 0 |  |
| 183 | 0 | 24.50 | 0 | 1.00 | 2 |  |
| 183 |  |  |  |  |  |  |
| 184 | 0 | 33.86 | 0 | .00 | 1 |  |
| 184 |  |  |  |  |  |  |
| 184 |  |  |  |  |  |  |
| 185 | 0 | 34.50 | 0 | .00 | 2 |  |
| 185 | 0 | 34.70 | 0 | .00 | 2 |  |
| 185 |  |  |  |  |  |  |
| 186 | 0 | 23.80 | 0 | 1.00 | 0 |  |
| 186 | 0 | 21.40 | 0 | 1.00 | 1 |  |
| 186 |  |  |  |  |  |  |
| 187 | 0 | 28.70 | 1 | .00 | 1 |  |
| 187 | 0 | 29.20 | 1 | .00 | 0 |  |
| 187 |  |  |  |  |  |  |
| 188 | 0 | 25.10 | 0 | 2.00 | 2 |  |
| 188 | 0 | 24.80 | 0 | 2.00 | 2 |  |
| 188 |  |  |  |  |  |  |
| 189 | 0 | 24.70 | 1 | .00 | 0 |  |
| 189 | 0 | 23.40 | 1 | 1.00 | 0 |  |
| 189 |  |  |  |  |  |  |
| 190 | 0 | 25.50 | 1 | .00 | 2 |  |
| 190 |  |  |  |  |  |  |
| 190 |  |  |  |  |  |  |
